# Targeting DNA repair mechanisms in cancer therapy: the role of small molecule DNA repair inhibitors

**DOI:** 10.1093/narcan/zcaf040

**Published:** 2025-11-03

**Authors:** Seula Jeong, Yuheon Chung, Soomin Heo, Kyungjae Myung

**Affiliations:** Center for Genomic Integrity, Institute for Basic Science, Ulsan 44919, Republic of Korea; Center for Genomic Integrity, Institute for Basic Science, Ulsan 44919, Republic of Korea; Center for Genomic Integrity, Institute for Basic Science, Ulsan 44919, Republic of Korea; Department of Biomedical Engineering, Ulsan National Institute of Science and Technology, Ulsan 44919, Republic of Korea; Center for Genomic Integrity, Institute for Basic Science, Ulsan 44919, Republic of Korea; Department of Biomedical Engineering, Ulsan National Institute of Science and Technology, Ulsan 44919, Republic of Korea

## Abstract

Genomic instability and the accumulation of DNA damage are hallmarks of cancer, often resulting from defects in DNA repair pathways. While normal cells rely on highly coordinated DNA damage response (DDR) mechanisms to maintain genomic integrity, cancer cells exploit aberrant DDR regulation to sustain uncontrolled proliferation and survival. Despite significant advancements in chemotherapy, targeted therapy, and immunotherapy, the emergence of resistance remains a major challenge in cancer treatment. Small molecule inhibitors targeting key DDR proteins have emerged as promising therapeutic agents, not only as direct anticancer drugs but also as indispensable tools for dissecting the molecular intricacies of DNA repair. Recent therapeutic approaches leverage synthetic lethality and DDR pathway vulnerabilities to selectively eradicate tumor cells while minimizing damage to normal tissues. These inhibitors provide insights into mechanisms of tumor resistance, facilitating the rational design of combination therapies to enhance treatment efficacy. This review examines the latest advancements in DNA repair-targeted therapeutics, with a focus on small molecule inhibitors currently under clinical investigation. Additionally, we discuss emerging strategies for optimizing DDR-targeted interventions, including biomarker-driven patient selection and rational drug combinations. Understanding these molecular interactions will contribute to the development of novel, more effective treatment paradigms for cancer therapy.

## Graphical Abstract

**Graphical Abstract:**
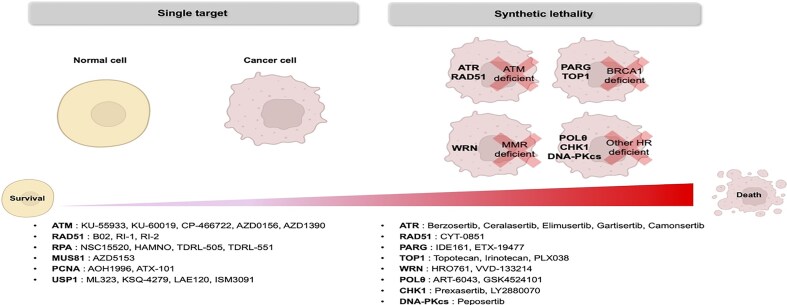

## Introduction

One of the hallmark features of cancer is genomic instability caused by defects in DNA damage repair mechanisms, which fundamentally drives uncontrolled cell division and proliferation. Normal cells maintain genomic stability through various DNA repair pathways, but cancer cells often exhibit defects in one or more repair pathways, leading to mutation accumulation and exacerbated genomic instability [1]. This instability not only contributes to cancer progression and metastasis but also serves as a major factor in treatment resistance.

**Figure 1.**
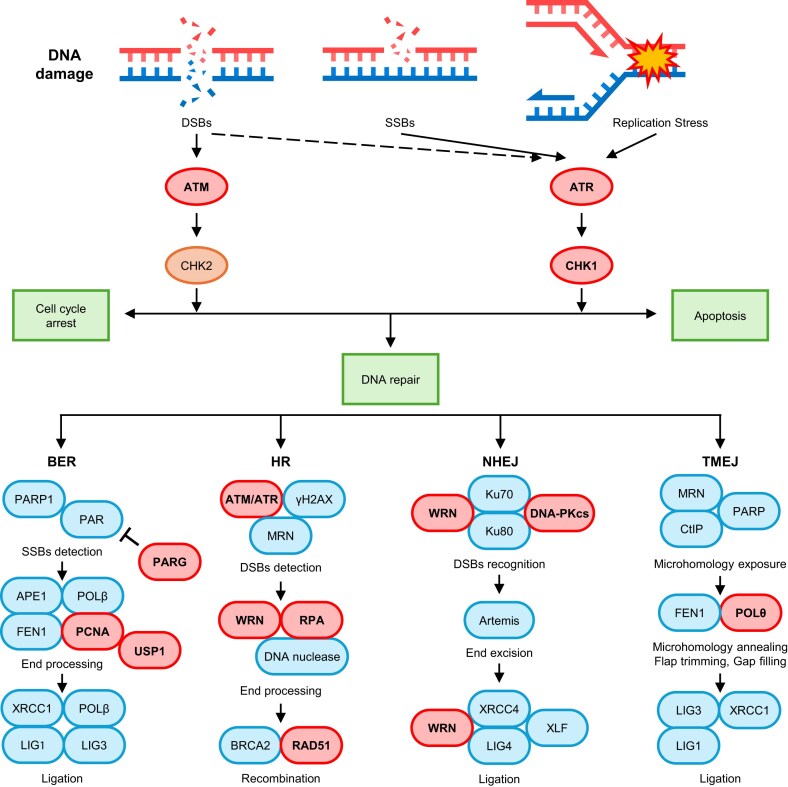
Overview of DDR and repair pathways DNA damage, including DSBs, SSBs, and replication stress, activates key sensor kinases such as ATM and ATR. ATM primarily responds to DSBs and signals through CHK2 to promote cell cycle arrest and facilitate DNA repair, while ATR responds to SSBs and replication stress via CHK1, leading either to repair or apoptosis depending on the extent of damage. DNA repair is orchestrated through four major pathways: BER, HR, NHEJ, and TMEJ. BER primarily addresses SSBs through PARP1 activation and subsequent recruitment of repair factors such as APE1, POLβ, and PCNA, with regulation by PARG. HR is a high-fidelity repair mechanism for DSBs involving ATM/ATR, γH2AX, MRN complex, RPA, WRN, and RAD51-mediated recombination. NHEJ repairs DSBs via Ku70/Ku80 heterodimer recognition, DNA-PKcs activation, and processing by Artemis, XRCC4, LIG4, and XLF, with WRN supporting intermediate processing. TMEJ, an alternative end-joining pathway mediated by POLθ and FEN1, repairs DSBs in the absence of classical HR or NHEJ. Proteins highlighted in red indicate key therapeutic targets currently under clinical trial for cancer treatment.

Base excision repair (BER) is a fundamental pathway that resolves small base lesions and abasic sites to maintain genomic stability (Fig. 1). The BER process requires the coordinated activity of proteins such as poly (ADP-ribose) glycohydrolase (PARG), proliferating cell nuclear antigen (PCNA), and ubiquitin-specific protease 1 (USP1), which regulate repair efficiency and ensure proper coordination with DNA replication and chromatin remodeling. Defects in BER compromise cellular responses to endogenous and exogenous DNA damage and contribute to tumorigenesis, highlighting BER-associated proteins as potential therapeutic targets [2].

Homologous recombination (HR) is an essential pathway for accurately repairing DNA double-strand breaks (DSBs) by using a sister chromatid with identical sequence as a template [3] (Fig. 1). The HR process requires the coordinated action of proteins such as breast cancer type 1/2 (BRCA1/2) and RAD51 homolog 1 (RAD51). Dysfunction of HR results in increased genomic instability and altered sensitivity to certain therapies [4]. Consequently, strategies that directly inhibit HR or exploit HR deficiencies as therapeutic vulnerabilities have gained significant attention as anticancer therapies [5].

Nonhomologous end joining (NHEJ), on the other hand, rapidly repairs DSBs by directly ligating broken DNA ends without requiring extensive sequence homology [6] (Fig. 1). NHEJ is particularly active during the G0/G1 phases of the cell cycle and serves as a primary DSB repair mechanism in situations requiring swift repair [7]. Cancer cells with defective HR often rely on alternative pathways like NHEJ for survival, making key NHEJ components attractive targets for selectively killing cancer cells [8].

Theta-mediated end joining (TMEJ), also known as polymerase TMEJ, serves as an alternative end-joining pathway that repairs DSBs when HR or classical NHEJ are unavailable (Fig. 1). This pathway is critically dependent on DNA polymerase theta (POLθ), which facilitates microhomology-mediated repair at resected DNA ends. Because cancer cells deficient in HR often rely on TMEJ for survival, POLθ has emerged as an attractive synthetic-lethal target in HR-deficient tumors [9].

The synthetic lethality is a promising concept in cancer biology that is being applied in clinical trials: loss of function in one gene alone does not significantly affect cell viability, but the simultaneous loss of two specific gene functions results in cell death [10]. In cancer therapy, targeting a specific defect in tumor cells (e.g. HR deficiency due to BRCA mutations) and pharmacologically inhibiting its compensatory pathway [e.g. poly-ADP ribose polymerase (PARP)-mediated opportunistic repair] can induce a “lethal vulnerability” absent in normal cells, selectively killing cancer cell [4, 11]. The remarkable efficacy of PARP inhibitors (PARPis) in BRCA-mutated cancers is a prime example, sparking extensive research and applications of synthetic lethal interactions among various DNA repair pathways [12].

This review summarizes key regulatory factors of HR and NHEJ pathways and anticancer strategies targeting them, while exploring the latest cancer treatment approaches based on synthetic lethality. Focusing on preclinical and clinical research trends of HR and NHEJ inhibitors, as well as clinical trial data for DNA damage repair targets, we examine the present and future of precision-targeted therapies for cancer patients with DNA repair deficiencies.

### Targeting key regulators of HR and NHEJ pathways in cancer

#### ATM

Ataxia-telangiectasia mutated protein (ATM) is a key kinase that recognizes DSBs and phosphorylates various downstream proteins involved in DNA repair, cell cycle regulation, and apoptosis [13]. When DSBs occur due to radiation or other insults, ATM activation induces an effective DNA damage response (DDR), making it a critical factor in cancer cell resistance to radiation [14]. Thus, ATM inhibitors are actively studied to impair cancer cell DNA repair, disrupt cell cycle checkpoints, and enhance sensitivity to radiotherapy or chemotherapy [15].

Small molecule ATM inhibitors almost universally target the ATP-binding pocket of the kinase domain [16] (Table 1). The classic first-generation inhibitor KU-55933 was followed by second generation compounds that improved potency, selectivity, and solubility, most notably KU-60019 [17, 18]. KU-60019 radiosensitizes cells at low micromolar concentrations and suppresses prosurvival signaling in glioma models [19, 20]. While it is nontoxic *in vitro* and safe after direct intracranial injection, its poor blood–brain barrier (BBB) permeability limits systemic use [19].

**Table 1.** HR and NHEJ pathway inhibitors in clinical trials.

Target protein	Drug	NCT identifier	Type of cancer	Combination	Phase	References
ATM	KU-55933	Preclinical	–	–	–	[17]
	KU-60019	Preclinical	–	–	–	[17–20]
	CP-466722	Preclinical	–	–	–	[21]
	AZD0156	ClinicalTrials.gov, NCT02588105	Advanced cancer	Olaparib or Irinotecan, FOLFIRI	1	[23, 24]
	AZD1390	ClinicalTrials.gov, NCT03423628	Brain cancer	Radiotherapy	1	[26, 27]
		ClinicalTrials.gov, NCT05182905	Glioma	–	1	[28]
		ClinicalTrials.gov, NCT06894979	Glioma	Radiotherapy	1	[29]
RAD51	B02	Preclinical	–	–	–	[36–39]
	RI-1	Preclinical	–	–	–	[40–42]
	RI-2	Preclinical	–	–	–	[43]
RPA	NSC15520	Preclinical	–	–	–	[51, 52]
	HAMNO	Preclinical	–	–	–	[53, 54]
	TDRL-505	Preclinical	–	–	–	[56, 57]
	TDRL-551	Preclinical	–	–	–	[58]
MUS81	AZD5153	ClinicalTrials.gov, NCT03205176	Lymphoma and solid tumor	Olaparib	1, 2	[61, 62, 65, 66]
PCNA	AOH1996	ClinicalTrials.gov, NCT05227326	Advanced solid tumor	–	1	[69, 71–73]
	ATX-101	ClinicalTrials.gov, NCT04814875	Ovarian cancer	Carboplatin or Pegylated liposomal doxorubicin	1, 2	[74, 76, 77]
		ANZCTR, ACTRN 12618001070224	Non-Hodgkin lymphoma	Etoposide or Prednisone, Cyclophosphamide, Doxorubicin, Rituximab	1, 2	[74]
USP1	ML323	Preclinical	–	–	–	[82–84]
	KSQ-4279 (RO7623066)	ClinicalTrials.gov, NCT05240898	HRD and advanced solid tumor	Olaparib or Carboplatin	1	[81, 85–87]
	LAE120	–	Advanced solid tumor	Olaparib	1	[88]
	ISM3091 (XL309)	ClinicalTrials.gov, NCT05932862	Advanced solid tumor	Olaparib	1	[89]

CP-466722 was an early ATP-competitive probe that rapidly and reversibly inhibited ATM. Kinome profiling revealed substantial off-target activity (25 kinases, including ALK2), poor selectivity, and a short half-life, leading to its discontinuation from clinical development [21]. Nevertheless, it remains useful in preclinical settings for transient ATM blockade.

AZD0156 (AstraZeneca) is an oral, ATP–competitive ATM inhibitor that showed strong synergy with PARPi and topoisomerase (TOP)–I inhibitors in BRCA–mutated xenografts and sensitized TP53–deficient lung–cancer cell lines to radiation [22, 23]. However, a phase I trial (NCT02588105) was terminated after only 2 of 46 patients achieved partial responses and hemolytic toxicity was observed [24].

In response, AstraZeneca advanced AZD1390, a next–generation, BBB–penetrant ATM inhibitor engineered to evade P-glycoprotein (P-gp/*ABCB1*)–mediated efflux—the ATP-driven export of drugs and other exogenous compounds from cells. AZD1390 achieves ∼6–fold higher brain exposure than AZD0156 and potently blocks ATM autophosphorylation and downstream signaling at 3 nM, inducing apoptosis when combined with radiation [25]. In orthotopic glioma models, AZD1390 prolongs survival, and PET imaging confirms its central nervous system (CNS) penetration [26]. Interim phase I data in recurrent glioblastoma (NCT03423628, NCT05182905, and NCT06894979) show a median overall survival of 12.7 months, suggesting clinical benefit [26–29]. Beyond radiosensitization, AZD1390 elicits immunomodulation by promoting cytosolic DNA accumulation, activating the cGAS–STING pathway, up–regulating PD–L1, and down–regulating galectin–9, thereby enhancing antitumor immunity [30]. Ongoing trials are testing AZD1390 in combination with radiotherapy, PARPi, or immunotherapies.

#### RAD51

RAD51 is a central protein in HR, forming filaments on single-stranded DNA (ssDNA) to facilitate strand exchange with homologous double-stranded DNA, enabling accurate DSB repair [31]. Overexpression of RAD51 in various cancers is associated with enhanced DNA repair capacity and resistance to radiation and chemotherapy [32]. RAD51 inhibition disrupts HR, forcing cells to rely on error-prone repair mechanisms and promoting genomic instability in replicating cancer cells [33].

Small–molecule inhibitors target RAD51 by blocking DNA binding, preventing oligomerization, or destabilizing filament assembly [34]. Unlike PARPi or ATM inhibitors that act upstream of the DDR, RAD51 inhibitors directly abrogate the core recombination step [31]. Tumors with elevated RAD51, high replication stress, or defective checkpoints are particularly vulnerable, whereas normal cells, which depend less on RAD51, may be spared—offering a potential therapeutic window [35].

B02 was identified from a high–throughput screen of >200 000 compounds [36] (Table 1). B02 binds the ATP–binding pocket of RAD51 and inhibits its ATP binding-dependent strand–exchange activity [37]. In cancer cells it suppresses RAD51 foci and sensitizes them to DNA–damaging agents such as cisplatin [38]. In xenograft studies, the combination of B02 and cisplatin markedly reduced tumor growth, demonstrating that RAD51 inhibition can potentiate chemotherapy–induced damage [39]. Although derivatives with improved affinity, pharmacokinetics, and activity against triple–negative breast cancer (TNBC) that was resistant to olaparib have been generated, B02 remains limited by hydrophobicity, low target specificity, and poor *in vivo* stability, and it has not entered clinical trials.

RI–1 was identified in a 10 000–compound screen [40]. RI–1 covalently modifies cysteine 319 at the RAD51 oligomerization interface, disrupting filament assembly and HR [40]. It increases DNA damage and enhances radiosensitivity and PARPi efficacy [41, 42]. However, chemical instability and a short half–life preclude *in vivo* use [43]. Its non–covalent analog RI–2 was designed for better metabolic stability but shows reduced potency [43]. Neither compound has progressed to human studies, yet they validate RAD51 as a tractable target.

Furthermore, RAD51 inhibitors are linked to synthetic lethality strategies (see Section 2.1), with expectations of selective killing in tumors with increased RAD51 dependency due to ATM or CHK1 deficiencies.

#### RPA

Replication protein A (RPA) is a heterotrimeric complex that binds ssDNA with high affinity and plays a central role in DNA replication and repair [44]. By stabilizing ssDNA intermediates and coordinating the recruitment of diverse repair factors, RPA is essential for multiple DDR pathways, including HR, BER, and nucleotide excision repair (NER) [45]. Notably, RPA subunits are often overexpressed or hyper-phosphorylated in cancers, correlating with enhanced replication stress tolerance and resistance to therapy [46]. Given its central role in preserving genome stability, RPA has emerged as an attractive target for cancer therapy, particularly in tumors exhibiting high replication stress or DNA repair deficiencies [47].

Several small molecule RPA inhibitors have been developed to either block RPA’s DNA-binding activity or interfere with its protein–protein interaction domains, though most candidates remain in preclinical stages of development [48]. Unlike upstream DDR modulators such as PARP or ATM inhibitors that act on signaling pathways, RPA inhibitors directly cripple the core ssDNA scaffolding required for replication and repair [49]. Tumors experiencing heightened replication stress or lacking robust checkpoints (e.g. due to ATR or CHK1 deficiencies) are particularly vulnerable to RPA suppression, whereas normal cells with lower replication stress may better tolerate transient RPA inhibition, providing a potential therapeutic window [50].

NSC15520 (fumaropimaric acid) was one of the first RPA inhibitors identified via screening efforts [51] (Table 1). It binds to the N-terminal domain of the RPA1 subunit (RPA70N), a region responsible for recruiting ATR and other proteins [51]. In cancer cell models, NSC15520 treatment destabilizes replication forks and sensitizes cells to genotoxic stress, effectively lowering the threshold for DNA damage-induced cell death [52]. However, NSC15520 is limited by suboptimal drug-like properties—including hydrophobicity, low specificity and poor metabolic stability—which have prevented its advancement beyond preclinical studies [52]. Derivatives with improved binding affinity and cellular uptake have been explored, but thus far none have progressed to clinical trials [52].

Another RPA70N-targeted inhibitor, HAMNO, similarly binds the RPA1 N-terminal domain and was shown to prevent ATR autophosphorylation [53]. While HAMNO increases DNA damage and can enhance the cytotoxicity of DNA crosslinking agents *in vitro*, its chemical instability and short half-life in cells have precluded *in vivo* use [54]. Like NSC15520, HAMNO remains a prototype tool compound, underscoring the challenges of targeting RPA’s protein-interaction domain [55].

Efforts have focused on inhibiting RPA’s DNA-binding activity. TDRL-505 was discovered through high-throughput screening as a small molecule that disrupts the binding of RPA to ssDNA [56]. TDRL-505 targets the central OB-fold domains of RPA1, preventing RPA from properly coating and protecting ssDNA. In cancer cells, this leads to unprotected replication forks, triggering replication stress, S-phase arrest, and apoptosis [56]. Importantly, TDRL-505 was found to synergize with DNA-damaging treatments: combining TDRL-505 with chemotherapeutic agents like cisplatin or etoposide markedly increases tumor cell killing compared to either agent alone [56, 57]. Structure–activity refinement of TDRL-505 yielded a second-generation analog, TDRL-551, with enhanced potency against RPA–ssDNA interactions [58]. TDRL-551 exhibits modest single-agent anticancer activity in preclinical models but shows strong synergy in combination with DNA-damaging chemotherapy, further validating the strategy of chemically targeting RPA’s ssDNA-binding function [58].

#### MUS81

Crossover junction endonuclease MUS81 (MUS81) is a structure specific endonuclease that resolves stalled replication forks by cleaving complex DNA intermediates such as Holliday junctions [59]. Its activity is essential for replication restart and repair, especially in cancer cells where genomic instability drives heightened replication stress [60]. Elevated MUS81 correlates with aggressive tumor behavior and poor prognosis; in gastric cancer, for instance, overexpression associates with metastasis and adverse outcomes [61] (Table 1). Recent evidence also links MUS81 to the regulation of epithelial–to–mesenchymal transition (EMT) transcription factors, notably ZEB1, suggesting a dual role in DNA repair and metastatic progression [61, 62].

No direct MUS81 inhibitors have entered clinical use, but indirect strategies are being explored [63]. A promising avenue targets the epigenetic regulator bromodomain-containing protein 4 (BRD4) [61]. The bromodomain inhibitor AZD5153 downregulates MUS81 transcription in cancer cells [61]. Although AZD5153 is not a nuclease inhibitor, its broad modulation of chromatin architecture alters the expression of numerous genes, including MUS81 and the EMT factor ZEB1 [61, 62]. In xenograft models, AZD5153 markedly inhibited metastatic spread—a phenotype recapitulated by MUS81 knockout (KO), confirming MUS81 as a key mediator of the drug’s activity [61]. Consequently, AZD5153 induces accumulation of unresolved DNA intermediates, enhances replication stress, and simultaneously suppresses cell motility and invasiveness [62]. UNI66, another inhibitor of BRD4, suppresses HR by inhibiting BRD4-mediated transcription of CtBP-interacting protein (CtIP) and RAD51, thereby inducing synthetic lethality in PARP1-deficient cells and enhancing tumor sensitivity to PARPis [64].

AZD5153 combined with olaparib is being evaluated as monotherapy in patients with solid tumors and hematologic malignancies (NCT03205176) [65]. It is orally bioavailable and has shown a favorable safety profile in early–phase trials. In hepatocellular carcinoma and other malignancies, AZD5153 downregulates oncogenic transcription programs, including c–Myc, producing pronounced antiproliferative and pro–apoptotic effects [66]. Clinical data confirm on–target engagement through suppression of BRD4–regulated genes [65]. Its impact on MUS81 and ZEB1 expression offers potential biomarker–guided therapeutic opportunities, particularly for tumors with high levels of these proteins [66].

#### PCNA

PCNA is a trimeric protein that acts as a clamp during DNA replication and repair, encircling the DNA double helix to recruit and anchor DNA polymerases and repair proteins for efficient function [67]. In rapidly dividing or treatment-resistant cancer cells, PCNA expression and activity are often elevated, reflecting high demands for DNA replication and damage repair [68]. Previously considered “undruggable” due to its essential role in normal cell survival, tumor–specific post–translational modifications generate a cancer–associated PCNA isoform with unique surface epitopes that can be selectively targeted [69, 70].

AOH1996 is a small–molecule inhibitor developed at City of Hope, named after a pediatric cancer patient [71] (Table 1). It binds at the trimer–trimer interface, locking PCNA in an inactive conformation and preventing interaction with client proteins [71]. Crystal structures reveal that three AOH1996 molecules occupy two PCNA trimers, effectively blocking function [71]. The resulting replication fork stalling, heightened replication stress, and transcription–replication conflicts produce DSBs and trigger apoptosis [71]. Preclinical data show activity in >70 solid–tumor cell lines (e.g. breast, lung, colorectal, prostate, etc.) with negligible effect on normal cells [72, 73]. AOH1996 is currently in a phase I trial for advanced solid tumors (NCT05227326).

ATX–101 is a cell–penetrating peptide from APIM Therapeutics that incorporates the natural PCNA–binding motif APIM (AlkB homolog 2 PCNA–interacting motif) [74, 75]. Normal cells, which rely primarily on PIP–box interactions under basal conditions, are less affected [76]. Preclinical studies demonstrate that ATX–101 induces apoptosis and markedly potentiates platinum drugs, gemcitabine, and radiotherapy by inhibiting DNA–repair pathways in tumor cells while sparing normal tissue [76, 77]. A phase I trial (ACTRN12618001070224) administered 20 terminal patients intravenously; 70 % achieved disease stabilization at 6 weeks, and one patient-maintained stability for >2 years [74]. No dose–limiting toxicities occurred up to 60 mg/m², with only mild infusion reactions. Phase II studies are underway (NCT04814875). Pharmacokinetics reveal a plasma half–life of <30 min, but weekly dosing sustains intracellular PCNA inhibition [74].

#### USP1

USP1 inhibitors are emerging precision anticancer agents targeting DDR pathways [78]. USP1 deubiquitinates PCNA and FANCD2—key mediators of replication-stress tolerance and interstrand crosslink (ICL) repair via the FANCI–FANCD2 axis [79]. At stalled forks, monoubiquitinated PCNA recruits translesion polymerases, while FANCD2-Ub localizes to damage sites to initiate ICL repair complex assembly; the USP1–UAF1 complex removes these ubiquitin marks to terminate signaling and resume replication/repair [80]. BRCA1-deficient tumors that depend on fork protection show synthetic lethal sensitivity, and high USP1 expression may further predict responsiveness to DDR-targeted strategies [81].

The preclinical inhibitor ML323 selectively targets USP1–UAF1, increases PCNA-Ub and FANCD2-Ub, amplifies replication stress, promotes apoptosis, and sensitizes tumor cells to cisplatin, suggesting a route to overcome platinum resistance [82–84]. KSQ-4279 (RO7623066), the first oral USP1 inhibitor to enter clinical trials, shows high selectivity and robust efficacy, including tumor regression in HR-deficient and PARPi-resistant models; combination with olaparib is synergistic (NCT05240898) [81, 85–87]. Ongoing phase I studies are evaluating monotherapy and combinations, with early readouts indicating favorable PK/PD and manageable, primarily hematologic, toxicity, supporting dose optimization and safety monitoring [86].

Additional agents include LAE120 (Laekna Inc.), an oral small molecule that has entered a first-in-human phase I study for advanced solid tumors in the U.S. The trial aims to evaluate safety, tolerability, pharmacokinetics, and preliminary efficacy, including potential combinations with PARPis [88]. As of 2025, the study has been initiated, although a ClinicalTrials.gov identifier has not yet been publicly listed. Another agent, ISM3091 (XL309), is being investigated in a U.S. phase I study (NCT05932862) for BRCA-mutated ovarian and breast cancers [89]. ISM3091 induces replication stress and DNA damage accumulation, and PARPi combinations are under active investigation [89]. Overall, USP1 inhibition offers a multifaceted strategy—synthetic lethality, fork destabilization, and reversal of PARPi resistance—and is poised to become a key component of future DDR-targeted combination therapies.

### Synthetic lethality-based strategies in cancer therapy

#### ATR and RAD51 inhibition strategies in ATM-deficient cancer cells

ATM-deficient cancer cells cannot effectively signal DSBs, making them highly dependent on ataxia telangiectasia and Rad3-related protein (ATR) to manage replication stress and DNA damage [90]. ATR is the principal regulator of replication-stress responses: when forks stall during S phase, ATR stabilizes forks, activates checkpoint kinase 1 (CHK1) to pause the cell cycle, and promotes repair [91]. Because oncogene-driven tumors experience chronic replication stress, they often become dependent on ATR signaling, whereas normal cells tolerate partial ATR inhibition even though complete ATR loss is lethal [92]. Small molecule ATR inhibitors block the kinase domain and prevent phosphorylation of substrates such as CHK1, leading to unchecked origin firing, fork collapse, S/G2 checkpoint abrogation, and replication catastrophe with widespread DSBs and cell death [93]. Tumors with ATM loss, p53 loss, or ARID1A mutation are therefore hypersensitive, making ATR an attractive synthetic lethal target [90, 94].

Berzosertib (M6620, formerly VX-970) is the first ATR inhibitor to enter clinical trials and is an intravenous, selective ATP-competitive agent [95]. In preclinical models it potentiated cisplatin and irradiation, particularly in ATM-deficient settings, by preventing G2 arrest and repair and forcing lethal mitosis; combinations with cisplatin or topotecan produced deeper tumor regressions than chemotherapy alone [95–97]. Early trials using day-2/9 dosing every 21 days showed manageable toxicity and activity; with topotecan, a recommended phase II dose of 210–240 mg/m^2^ yielded partial responses or durable stable disease in small cell lung and ovarian cancers (NCT02157792) [95, 96, 98]. A notable complete response occurred with monotherapy in ATM-mutant gastric cancer, illustrating synthetic lethality. Myelosuppression—especially neutropenia in chemotherapy combinations—is dose limiting; in platinum-resistant ovarian cancer, berzosertib plus gemcitabine produced responses in ∼17% but required dose reductions. A randomized study with topotecan in relapsed small cell lung cancer did not significantly improve progression-free survival (NCT03896503), underscoring the need for biomarker-driven combinations and patient selection [96, 99, 100].

Ceralasertib (AZD6738) is an orally bioavailable, selective ATR inhibitor optimized from AZ20 for potency and pharmacokinetics [101]. It enhances DNA damaging treatments such as carboplatin in xenografts and is advancing through phase I/II trials [102, 103]. Toxicities are mainly hematologic and gastrointestinal; cardiotoxicity seen at high mouse doses has not emerged as a dominant clinical signal [104]. Because ATR inhibition can increase cytosolic DNA and activate cGAS–STING, ceralasertib is being combined with immunotherapy [105]. Preclinical hepatocellular carcinoma models showed improved tumor control with anti-PD-L1 therapy, and in the ATLANTIS phase II study in PD-1–refractory melanoma, durvalumab plus ceralasertib achieved objective responses in a subset of patients (∼10%–15%, NCT03780608) [106–108]. A parallel gastric-cancer study is ongoing (NCT03682289), supporting ATR inhibitors as both chemo/radiation sensitizers and immune modulators [108].

Elimusertib (BAY 1895344) is a potent oral ATR inhibitor with strong target engagement in preclinical models and a favorable nonclinical safety profile [109–112]. Intermittent schedules (3 days on/4 days off; 40 mg twice daily) permit normal-tissue recovery [109]. Single-agent activity has been observed in ATM-deficient tumors, including lymphomas; in a first-in-human study, 30% of evaluable patients achieved ≥4-month stable disease and two patients with ATM-loss cancers achieved partial responses (NCT03188965) [109]. Ongoing studies focus on ATM-mutant chronic lymphocytic leukemia, lymphomas, and solid tumors [113].

Additional ATR inhibitors include gartisertib (M4344/VX-803), which is being combined with the PARPi niraparib to co-target replication stress and single-strand break repair (NCT04149145) [114], and camonsertib (RP-3500), a next generation oral agent developed via a synthetic lethality platform [115]. Early studies of camonsertib reported partial responses—particularly in ATM-loss and high replication stress tumors—using intermittent dosing with manageable anemia and thrombocytopenia (NCT04497116) [115]. Camonsertib is under evaluation alone and with PARPi such as talazoparib to overcome PARPi resistance (NCT04972110) [116]; Roche licensed the program in 2023, and phase II cohorts emphasize biomarker-selected populations.

ATM-deficient cancer cells rely heavily on RAD51-mediated HR because DSB signaling is weakened, and CHK1-deficient cells similarly depend on RAD51 due to impaired checkpoints [117]. RAD51 inhibition can therefore drive selective lethality by amplifying unrepaired DNA damage in these backgrounds [42, 118].

CYT-0851 is the most advanced clinical candidate nominally targeting RAD51 and is being evaluated orally as monotherapy and with chemotherapies such as gemcitabine or capecitabine (NCT03997968) [119]. Preclinically, it reduces RAD51 foci, increases γH2AX, and synergizes with PARPis in BRCA-proficient TNBC [120]. In first-in-human studies, it showed good oral bioavailability and a manageable safety profile; among evaluable patients, partial responses and durable disease stabilization were observed. Mechanistic work suggests CYT-0851 may act primarily as an MCT1 inhibitor, causing metabolic stress that secondarily impairs HR; despite this, it remains a promising way to exploit RAD51 dependence [121].

Inhibiting RAD51 pathway function can broaden the utility of PARPi in BRCA-wild-type tumors by suppressing HR and creating synthetic lethality [122]. The concept of “RAD51 addiction” in high replication stress cancers further supports combining RAD51 pathway agents with chemotherapy or other DDR inhibitors [123].

#### POLθ, CHK1, and DNA-PKcs inhibition strategies in HRD cancers

HR-deficient (HRD) cancer cells, such as those with BRCA1/2 mutations, lose the ability to accurately repair DSBs and rely on error-prone repair pathways [124]. POLθ, encoded by *POLQ* gene, is a specialized polymerase–helicase that mediates TMEJ, an error-prone backup for DSB repair when HR is unavailable. POLθ functions as a last resort repair option for cells that cannot use HR or classical NHEJ, and BRCA1/2-mutant tumors become highly dependent on POLθ to repair replication-associated DSBs and rescue broken forks [124] (Table 2). POLθ overexpression correlates with genomic instability and poor outcomes, suggesting tumors exploit its error-prone repair [125]. The polymerase domain can insert across damaged bases and microhomologies, and the helicase-like N-terminal domain aligns DNA ends for joining [126]. POLθ inhibitors block polymerase activity to prevent TMEJ and selectively kill HR-defective cells while sparing HR-proficient normal cells [127].

**Table 2.** Clinical trials employing synthetic lethality strategies.

Type of cancer	Target protein	Drug	NCT identifier	Combination	Phase	References
ATM-deficient cancer	ATR	Berzosertib (M6620, VX-970)	ClinicalTrials.gov, NCT02157792	Gemcitabine or Cisplatin, Etoposide, Carboplatin, Irinotecan	1	[95, 96]
			ClinicalTrials.gov, NCT03896503	Topotecan	2	[99]
		Ceralasertib (AZD6738)	ClinicalTrials.gov, NCT03780608	Durvalumab	2	[107, 108]
			ClinicalTrials.gov, NCT03682289	Monotherapy or Olaparib, Durvalumab	2	[108]
		Elimusertib (BAY1895344)	ClinicalTrials.gov, NCT03188965	–	1	[109]
		Gartisertib (M4344/VX-803)	ClinicalTrials.gov, NCT04149145	Niraparib	1	[114]
		Camonsertib (RP-3500)	ClinicalTrials.gov, NCT04497116	Monotherapy or Talazoparib, Gemcitabine	1, 2a	[115]
			ClinicalTrials.gov, NCT04972110	Niraparib or Olaparib	1b, 2	[116]
	RAD51	CYT-0851	ClinicalTrials.gov, NCT03997968	Gemcitabine or Capecitabine, Rituximab and bendamustine	1, 2	[119, 121]
HRD cancer	POLθ	ART-6043	ClinicalTrials.gov, NCT05898399	Olaparib or Niraparib	1, 2a	[124]
		GSK4524101	ClinicalTrials.gov, NCT06077877	Niraparib	1, 2	[130]
	CHK1	Prexasertib (LY2606368)	ClinicalTrials.gov, NCT03414047	–	2	[147]
			ClinicalTrials.gov, NCT03057145	Olaparib	1	[148]
			ClinicalTrials.gov, NCT02124148	Cisplatain or Cetuximab, G-CSF, Pemetrexed, Fluorouracil, Leucovorin, LY3023414	1	[149]
			ClinicalTrials.gov, NCT02808650	–	1	[150, 151]
		LY2880070	ClinicalTrials.gov, NCT02632448	Gemcitabine	1, 2	[153–155]
			ClinicalTrials.gov, NCT05275426	Gemcitabine	2	[155]
	DNA-PKcs	Peposertib (M3814)	ClinicalTrials.gov, NCT04533750	Cisplatin or Radiotherapy	1	[170]
			ClinicalTrials.gov, NCT03770689	Capecitabine or Radiotherapy	1, 2	[170, 171]
			ClinicalTrials.gov, NCT04092270	Pegylated liposomal doxorubicin	1	[161]
			ClinicalTrials.gov, NCT05687136	M1774 (ATR inhibitor)	1b	[172]
			ClinicalTrials.gov, NCT04068194	Avelumab	1, 2	[173]
BRCA-deficient cancer	PARG	IDE161	ClinicalTrials.gov, NCT05787587	Monotherapy or Pembrolizumab	1	[176, 181]
		ETX-19477	ClinicalTrials.gov, NCT06395519	–	1, 2	[176, 182]
	TOP1	Topotecan	ClinicalTrials.gov, NCT00484666	Docetaxel	1, 2	[188, 189]
			ClinicalTrials.gov, NCT00305942	Carboplatin	2	[190]
			ClinicalTrials.gov, NCT00276796	Paclitaxel or Cisplatin	2	[191]
			ClinicalTrials.gov, NCT03289910	Carboplatin or Veliparib	2	[197]
		Irinotecan/SN-38	ClinicalTrials.gov, NCT00311610	Liposomal SN-38	2	[192,193]
			ClinicalTrials.gov, NCT02292758	Cetuximab or Bevacizumab	2	[194]
			ClinicalTrials.gov, NCT00389870	Panitumumab or Cyclosporine	3	[195]
			ClinicalTrials.gov, NCT03290937	Utomilumab or Cetuximab	1	[196]
		PLX038 (PEGylated SN-38)	ClinicalTrials.gov, NCT05465941	–	2	[199]
			ClinicalTrials.gov, NCT04209595	Rucaparib or Ondansetron	1, 2	[198,200]
			ClinicalTrials.gov, NCT06337630	Tuvusertib (ATR inhibitor)	1	–
MMR-deficient cancer	WRN	HRO761	ClinicalTrials.gov, NCT05838768	Monotherapy or Pembrolizumab, Irinotecan	1	[205]
		VVD-133214	ClinicalTrials.gov, NCT06004245	Monotherapy or Pembrolizumab	1	[206]

In BRCA1/2-mutant cell lines, POLθ inhibition causes accumulation of unrepaired damage and apoptosis, mirroring POLQ KO [127, 128]. The tool compound ART558 inhibit POLθ’s extension activity in the nanomolar range, indicating specificity for TMEJ [129]. Combining ART558 with olaparib increases replication stress and produces synergistic lethality [128]. POLθ inhibition can also overcome PARPi resistance caused by loss of the 53BP1/Shieldin complex, because such tumors remain dependent on POLθ for residual end joining [128]. In xenograft models of BRCA-mutant breast cancer, POLθ inhibitor monotherapy suppressed tumor growth, and in 53BP1-deficient, HR-restored tumors, POLθ inhibition resensitized tumors to PARP inhibition [128]. These findings position POLθ inhibitors as synthetic lethal monotherapies for HRD cancers and as agents to address PARPi resistance.

ART-6043 is the oral POLθ inhibitor to enter clinical trials developed by Artios. ART-6043 entered global first-in-human studies in 2021 as monotherapy and in combination with PARPi such as olaparib or niraparib (NCT05898399) in BRCA1/2-mutant breast, ovarian, prostate, and pancreatic cancers [124, 129]. Interim results indicate target engagement, an acceptable safety profile dominated by low grade fatigue and nausea, and disease stabilization in some patients; the combination with olaparib or niraparib has shown at least additive antitumor activity [124, 129]. Based on phase I signals, Artios initiated a phase II expansion in BRCA-mutant breast cancer to test whether POLθ inhibition can deepen responses or overcome PARPi resistance [124, 129].

GSK-4524101 is a POLθ inhibitor being developed by GlaxoSmithKline. While details are limited (structure not public), GSK disclosed the start of a Phase I trial (NCT06077877) of GSK-4524101 in late 2023, in patients with advanced solid tumors harboring HRD [130]. Likely, GSK’s compound shares a similar mechanism—selective POL inhibition. Preclinically, GSK’s POLθ inhibitor demonstrated synergy with niraparib and killed BRCA KO cancer cells [130].

HRD cancer cells also heavily rely on CHK1, a key downstream effector of ATR that stabilizes stressed replication forks and enforces the S and G2/M checkpoints by inactivating CDC25 phosphatases [131–133]. In HRD cells, CHK1 inhibition permits division with unrepaired lesions, leading to mitotic catastrophe and apoptosis, and this effect is amplified in p53-deficient tumors that have lost the G1/S checkpoint [134].

Prexasertib (LY2606368) is an intravenous, potent ATP-competitive CHK1 inhibitor (IC_50_ < 1 nM) with weaker activity against CHK2 [135]. By disabling checkpoint arrest after DNA damage, it forces entry into mitosis with broken DNA, producing γH2AX foci, fragmented chromosomes, and apoptosis [136]; as a single agent it can also trigger DSBs via unrestrained origin firing, reflected by increases in RAD51 and RPA foci [137–139]. Tumor cells with p53 disruption or cyclin-E overactivity are particularly sensitive [140]. Preclinically, prexasertib is active in squamous carcinomas, small cell lung cancer, and ovarian cancer, often in RB-deficient or high-E2F tumors [140–142]. In head and neck xenografts it induced regressions, and in combination it synergized with cisplatin, gemcitabine, and PARPi [143–145]; in BRCA-proficient TNBC, prexasertib plus PARPi produced *in vivo* efficacy, suggesting utility in PARPi resistance [140, 142].

Clinically, phase I/II trials show manageable safety dominated by transient, dose limiting neutropenia (105 mg/m² IV every 14 days), while nonhematologic events are generally mild [141, 142, 146]. Objective responses have been observed: in platinum-resistant ovarian cancer, prexasertib monotherapy achieved ∼30% responses (including a complete response) irrespective of BRCA status, with some patients maintaining stable disease for 6–12 months (NCT03414047) [147]. Activity in recurrent BRCA-wild-type TNBC has been modest (NCT03057145 and NCT02124148), and a small study in small cell lung cancer combining prexasertib with an immune checkpoint inhibitor yielded responses in a subset (NCT02808650) [148–151].

LY2880070 is a second generation, orally bioavailable, selective ATP-competitive CHK1 inhibitor (IC_50_ ∼1 nM) with minimal CHK2 activity [152]. As monotherapy it showed limited efficacy despite pharmacodynamic target engagement (reduced phospho-CDC25C), possibly due to exposure or tumor heterogeneity.

In a phase I expansion in heavily pretreated pancreatic cancer, continuous oral LY2880070 combined with low dose weekly gemcitabine (~300 mg/m²) was tolerable, with neutropenia and fatigue as expected, and produced signs of disease stabilization in several patients (NCT02632448) [153–155]. Separately, NCT05275426 is a Phase II clinical trial evaluating the safety and preliminary efficacy of the oral CHK1 inhibitor LY2880070 in combination with low-dose intravenous gemcitabine for patients with relapsed or refractory Ewing sarcoma, Ewing-like sarcoma, or desmoplastic small round cell tumor (NCT05275426) [155].

DNA-dependent protein kinase (DNA-PK) is the core enzyme of NHEJ, the pathway that repairs DSBs by ligating broken ends [156]. DNA-PK comprises the catalytic subunit DNA-PKcs and the DNA-binding Ku70/80 heterodimer [157]. After a DSB, Ku binds DNA ends, recruits DNA-PKcs, and promotes its autophosphorylation to coordinate end processing and ligation [158]. DNA-PK is critical for DSB repair in G1, when HR is unavailable, and for simple breaks induced by ionizing radiation or TOP poisons [159–161]. Inhibiting DNA-PK blocks NHEJ, leaving DSBs unrepaired or diverting them to slower, error-prone backups such as TMEJ [162]. Therapeutically, DNA-PK inhibitors act as radio- and chemosensitizers: by preventing rapid DSB repair, they enhance killing by radiotherapy and DSB-inducing drugs [163, 164]. Tumors often rely on NHEJ and can be more sensitive to its loss than normal tissues, which can use HR in S/G2 [160]. In tumors, DNA-PK inhibition increases mis-repair and genomic instability beyond viability [165]. Combinations with PARPi or ATR inhibitors can be lethal because NHEJ and HR compensate for each other, especially in p53-null settings [162, 166]. ATM-deficient tumors are particularly reliant on DNA-PK, making DNA-PK inhibition a synthetic lethal partner for ATM loss [167].

Peposertib (M3814) is an orally bioavailable, highly selective DNA-PKcs inhibitor from Merck KGaA that binds the ATP active site with low nanomolar affinity [168]. It robustly sensitizes tumor cells and xenografts to radiation and chemotherapy; in colorectal and head and neck models, adding peposertib to fractionated radiation significantly delayed tumor growth versus radiation alone [168]. As expected for a nondamaging agent, monotherapy produced only modest delays, but combinations yielded persistent γH2AX, fragmented nuclei, and mitotic catastrophe [164, 168, 169].

In early clinical studies, peposertib has been combined with radiotherapy, chemoradiotherapy, and systemic chemotherapy. In locally advanced head and neck cancer, 5-day-per-week dosing with cisplatin–radiation was tolerated up to 150 mg/day, whereas some patients developed mucositis at 200 mg; complete responses were observed in certain HPV-negative patients (NCT04533750) [170]. Short course radiotherapy plus peposertib for rectal cancer improved tumor necrosis and pathologic responses, and another rectal cancer study using peposertib up to 150 mg/day with full dose capecitabine–radiation showed no unexpected toxicities, tumor downstaging in over half of patients, and some complete pathologic responses, albeit with slightly higher acute grade-3 lymphopenia (NCT03770689) [170, 171]. In recurrent ovarian cancer, peposertib (up to 250 mg once daily or 100 mg twice daily) with pegylated liposomal doxorubicin produced partial responses with manageable hematologic toxicity (NCT04092270) [161].

Rational combinations include ATR and DNA-PK inhibitors—such as peposertib with Merck’s ATR inhibitor M1774 in phase Ib (NCT05687136)—in which ATR blockade forces reliance on NHEJ that is then disabled by DNA-PK inhibition, and DNA-PK inhibitors with immunotherapy, because unrepaired DSBs can activate cGAS–STING and increase immunogenicity [172]. Trials of peposertib plus avelumab in solid tumors have been feasible with doses up to 400 mg twice daily and without increased immune-related adverse events (NCT04068194); early efficacy signals include prolonged stable disease in a microsatellite-stable (MSS) colorectal cancer patient [173].

#### PARG and TOP1 inhibition strategies in BRCA-deficient cancers

PARG is the primary enzyme that cleaves and removes poly (ADP-ribose) (PAR) chains from proteins, effectively reversing PARP activity [174]. After DNA damage, PARP1 adds PAR chains to itself and other proteins to signal repair, and PARG hydrolyzes these chains to reset repair complexes and recycle PARP [175]. Inhibiting PARG causes PAR to accumulate on chromatin and traps PARP–DNA complexes, which stalls repair; persistent PARylation can block factor turnover [176]. PARG inhibition also hyperactivates PARP and depletes NAD⁺, compounding repair deficits [177]. The net effect resembles PARP inhibition but via excess PAR rather than its absence [177]. Tumors, which often exhibit heightened PARP activity, rely on timely PAR turnover, so cancers with replication stress or HR defects may be vulnerable to PARG inhibitors, as PAR accumulation at forks can precipitate fork collapse [178]. PARG inhibition also creates synthetic lethal interactions distinct from PARPi; in some HR-proficient but replication-stressed settings PARG can be essential [179, 180]. Although PARG was long considered difficult to drug because its regulatory macrodomain binds PAR rather than presenting a classic small molecule pocket, recent advances have yielded compounds that occupy the ADP-ribose–binding groove [175].

In preclinical models of HRD cancers, including BRCA2-null patient-derived xenografts, IDE161 monotherapy produced marked growth inhibition or regression and retained efficacy in PARPi-resistant tumors [177]. By preventing PAR degradation, IDE161 induces replication stress with fork slowing, ssDNA gap accumulation, and transcription–replication conflicts that can progress to fork collapse and cell death [181]. Normal cells with intact checkpoints tolerate transient PAR accumulation, and IDE161 showed selective cytotoxicity toward DDR-defective cancer cells while sparing normal cells *in vitro* [181]. IDE161 is in phase I trials for BRCA1/2-mutated and HRD solid tumors (NCT05787587) [181].

ETX-19477 from 858 Therapeutics is a small molecule PARG inhibitor in phase I (NCT06395519) [176, 182]. ETX-19477 has low nanomolar cellular potency, induces PAR accumulation at damage sites, and shows broad antiproliferative activity—particularly in HRD or high replication stress models such as ER⁺/HER2^-^ breast, serous ovarian, lung, and gastric cancers [182]. It is orally bioavailable with favorable pharmacokinetics and was well tolerated in animals, achieving plasma levels that drove tumor PAR accumulation [182].

Another promising approach in BRCA-deficient tumors is increasing DNA replication stress through TOP1 inhibition [183]. TOP1 inhibitors stabilize the transient TOP1–DNA cleavage complex during replication, and collisions with advancing forks convert these single-strand lesions into DSBs that HRD cells cannot resolve, triggering apoptosis [184, 185]. The camptothecin derivatives topotecan and irinotecan (via the active metabolite SN-38) exemplify this class [186]. Topotecan, engineered with a water solubilizing dimethylaminomethyl side chain for intravenous and oral use, binds the TOP1–DNA complex, intercalates at the nick, and interacts with catalytic tyrosine 723 to lock the complex, producing fork-collision–induced DSBs [187]. Clinically, topotecan is used in ovarian cancer (NCT00484666) [188, 189], small cell lung cancer (∼20% response as second line, NCT00305942) [190], and cervical cancer (NCT00276796) [191], whereas irinotecan/SN-38 is a backbone drug in colorectal (NCT00311610 and NCT02292758) [192–194] and other solid tumors (NCT00389870 and NCT03290937) [195, 196]. Resistance mechanisms include increased drug efflux and TOP1 alterations that reduce binding or enhance religation. Ongoing trials (NCT03289910) continue to evaluate these agents and combinations in HRD [197].

PLX038, a PEGylated SN-38 formulation designed to improve pharmacokinetics and tumor targeting, enhances therapeutic efficacy in HRD, ATM- or BRCA-mutant tumors [198]. In preclinical models it produced tumor regressions and growth delays, and early phase I studies in solid tumors indicate that PLX038 can be combined with PARPi such as rucaparib with manageable toxicity, leveraging continuous DNA damage alongside HR blockade [198]. PLX038 is also being tested as monotherapy in platinum-resistant ovarian cancer (Phase II, NCT05465941) [199]. Combination trials with PARPi (NCT04209595) aim to maximize DNA-damage accumulation and achieve synthetic lethality [198, 200], and a reported case of ATM-mutated breast cancer treated with PLX038 plus a PARPi achieved sustained complete remission (NCT06337630).

#### WRN inhibition strategies in MMR-deficient cancers

Mismatch repair (MMR)–deficient tumors with high microsatellite instability (MSI-H) carry heavy mutational burdens and genomic instability, respond well to immunotherapy, and show strong dependence on Werner syndrome helicase (WRN) for coping with replication-associated DNA damage [201]. Because MMR defects increase replication stress, WRN, a RecQ-family enzyme with both helicase and exonuclease, becomes critical for maintaining genome stability, creating a synthetic lethal requirement in MSI-H cancers while remaining largely dispensable in MSS contexts [202]. In MSI-H settings, WRN loss triggers fork collapse, DSBs, mitotic catastrophe, and cell death, which makes WRN an attractive therapeutic target [201, 203, 204].

WRN inhibition is therefore being pursued as a selective synthetic lethality strategy for MSI-H tumors, with clinical evaluation of small molecule inhibitors such as HRO761 and VVD-133214 [205, 206]. HRO761, discovered by Novartis, is a first-in-class allosteric helicase inhibitor that binds a pocket at the D1–D2 interface, locks WRN in a closed, non-unwinding conformation, and does not compete with ATP [205]. It is highly potent and selective *in vitro* and, in cells, recapitulates WRN KO: MSI-H cancer cells accumulate γH2AX-marked breaks and undergo p53-independent cell death, while MSS cells are spared [205]. Notably, HRO761 induces WRN protein degradation specifically in MSI-H cells, likely reflecting ubiquitin–proteasome turnover of trapped, inactive WRN; this provides a useful pharmacodynamic marker of target engagement [205].

HRO761 is being tested in a Phase I/Ib trial (NCT05838768) in MSI-H advanced solid tumors as monotherapy and in combinations, including with the PD-1 antibody tislelizumab and with irinotecan [205, 207]. The drug is given orally on 28-day cycles. Early findings show pharmacodynamic activation and tumor regressions in some MSI-H patients, little activity in MSS tumors, and no dose limiting toxicities at initial exposure levels, which has enabled full dose combinations.

Roche/Genentech and Vividion have developed VVD-133214, a covalent allosteric inhibitor that targets cysteine 727 in the WRN helicase domain [206]. Binding is cooperative with nucleotide, allowing the compound to lock WRN in an inactive conformation through an irreversible bond [206]. Preclinical data show selective lethality in MSI-H cells, proteasomal degradation of bound WRN, and robust tumor regressions in multiple MSI-H xenograft and PDX models, with minimal effects in MSS controls [206]. Combination with irinotecan further enhanced tumor regressions, consistent with synergy between induced replication stress and WRN blockade [208]. As of late 2023, a first-in-human study had entered startup for MSI-H cancers, with oral dosing anticipated and safety monitoring focused on off-target covalent interactions; chemoproteomic discovery was used to maximize selectivity (NCT06004245) [206, 207].

These clinical programs represent the first translation of WRN synthetic lethality to the clinic and are expected to clarify the therapeutic value of WRN targeting in MSI-H cancers, either alongside or after immunotherapy.

## Conclusion

Defects in DDR are a defining vulnerability of many cancers and continue to reshape therapeutic strategy. By exploiting pathway interdependencies—most notably HR, NHEJ, and replication-stress signaling—agents such as PARP, ATR, POLθ, CHK1, PARG, TOP1, and WRN inhibitors are moving from mechanism to medicine. As reviewed here, these drugs can function as monotherapies in biomarker-selected tumors or deliver potent synergy with radiotherapy and cytotoxic chemotherapy by converting reparable lesions into lethal DNA damage. Beyond classic DDR targets, modality-expanding approaches are emerging: 2-chloro-*N,N*-diethylethanamine hydrochloride (CDEAH), a guanine-alkylating agent, preferentially kills PARP1-deficient cells by forcing dependence on BER and NER and shows combination potential with PARP inhibition [209]; and cancer-specific INDEL attacker (CINDELA) leverages CRISPR–Cas9 to inflict multi-site DSBs specifically at mutated sequences, offering a blueprint for truly personalized, genotype-directed cytotoxicity [210].

A central challenge is widening the therapeutic index. While cancer cells are often hyper-dependent on HR mediators such as RPA and RAD51 or replication checkpoints (e.g. ATR/CHK1) owing to oncogene-driven stress and checkpoint erosion, normal proliferating tissues still require baseline DDR, making myelosuppression and gastrointestinal toxicity recurring on-target effects. Strategies to enhance selectivity include (i) biomarker-based enrichment (e.g. BRCA/HRD, ATM loss, MSI-H for WRN inhibition); (ii) synthetic-lethality pairing to reduce dose intensity (e.g. ATR+PARP, POLθ+PARP, DNA-PKcs+radiation); (iii) intermittent/scheduled dosing to exploit tumor repair liabilities while allowing normal-tissue recovery; (iv) tumor-directed delivery and CNS-penetrant design where needed; and (v) immune-oncology combinations, as persistent DNA damage can activate cGAS–STING, increasing immunogenicity but also necessitating vigilance for immune-related adverse events. Long-term monitoring for selection of resistant clones, genomic instability in survivors, and secondary malignancies remains imperative as DDR-targeted regimens move earlier in care.

Looking forward, three forces will accelerate clinical impact: (i) Precision patient selection using composite biomarkers (genotype, functional HRD, replication-stress signatures, and dynamic pharmacodynamic readouts); (ii) Rational combinations that collapse compensatory repair (ATR with DNA-PKcs or POLθ; PARP with PARG or TOP1 payloads) while mitigating overlapping toxicities; and (ii) Next-generation modalities that expand beyond occupancy-based inhibition—such as targeted degraders, genome-editing approaches like CINDELA, and small molecules like CDEAH that exploit context-specific repair dependence. As clinical datasets mature, DDR-targeted therapies are poised to integrate into standard care pathways, improving survival and quality of life while advancing the promise of mechanism-guided, minimally toxic precision oncology.

## Data Availability

No new data were generated or analyzed in support of this research.

## References

[1] Hanahan D, Weinberg RA. Hallmarks of cancer: the next generation. Cell. 2011;144:646–74. 10.1016/j.cell.2011.02.013. 21376230

[2] Krokan HE, Bjørås M. Base excision repair. Cold Spring Harb Perspect Biol. 2013;5:a012583. 10.1101/cshperspect.a012583. 23545420 PMC3683898

[3] Wright WD, Shah SS, Heyer W-D. Homologous recombination and the repair of DNA double-strand breaks. J Biol Chem. 2018;293:10524–35. 10.1074/jbc.TM118.000372. 29599286 PMC6036207

[4] Farmer H, McCabe N, Lord CJ et al. Targeting the DNA repair defect in BRCA mutant cells as a therapeutic strategy. Nature. 2005;434:917–21. 10.1038/nature03445. 15829967

[5] Zhang J . The role of BRCA1 in homologous recombination repair in response to replication stress: significance in tumorigenesis and cancer therapy. Cell Biosci. 2013;3:11. 10.1186/2045-3701-3-11. 23388117 PMC3599463

[6] Lieber MR . The mechanism of double-strand DNA break repair by the nonhomologous DNA end-joining pathway. Annu Rev Biochem. 2010;79:181–211. 10.1146/annurev.biochem.052308.093131. 20192759 PMC3079308

[7] Mao Z, Bozzella M, Seluanov A et al. DNA repair by nonhomologous end joining and homologous recombination during cell cycle in human cells. Cell Cycle. 2008;7:2902–6. 10.4161/cc.7.18.6679. 18769152 PMC2754209

[8] Helleday T . The underlying mechanism for the PARP and BRCA synthetic lethality: clearing up the misunderstandings. Mol Oncol. 2011;5:387–93. 10.1016/j.molonc.2011.07.001. 21821475 PMC5528309

[9] Wyatt DW, Feng W, Conlin MP et al. Essential roles for polymerase θ-mediated end joining in the repair of chromosome breaks. Mol Cell. 2016;63:662–73. 10.1016/j.molcel.2016.06.020. 27453047 PMC4992412

[10] Bridges CB . The origin of variations in sexual and sex-limited characters. Am Nat. 1922;56:51–63. 10.1086/279847.

[11] Bryant HE, Schultz N, Thomas HD et al. Specific killing of BRCA2-deficient tumours with inhibitors of poly(ADP-ribose) polymerase. Nature. 2005;434:913–7. 10.1038/nature03443. 15829966

[12] Fong PC, Boss DS, Yap TA et al. Inhibition of poly(ADP-Ribose) polymerase in tumors from BRCAMutation carriers. N Engl J Med. 2009;361:123–34. 10.1056/NEJMoa0900212. 19553641

[13] Shiloh Y . ATM and related protein kinases: safeguarding genome integrity. Nat Rev Cancer. 2003;3:155–68. 10.1038/nrc1011. 12612651

[14] Bakkenist CJ, Kastan MB. DNA damage activates ATM through intermolecular autophosphorylation and dimer dissociation. Nature. 2003;421:499–506. 10.1038/nature01368. 12556884

[15] Ampolini EA, Jimenez-Sainz J, Long DT. The development of ATM inhibitors in cancer therapy. Targ Oncol. 2025;20:281–97. 10.1007/s11523-025-01136-6. PMC11933189 40024979

[16] Huang C, Filippone NR, Reiner T et al. Sensors and inhibitors for the detection of ataxia telangiectasia mutated (ATM) protein kinase. Mol Pharmaceutics. 2021;18:2470–81. 10.1021/acs.molpharmaceut.1c00166. PMC12930525 34125542

[17] Hickson I, Zhao Y, Richardson CJ et al. Identification and characterization of a novel and specific inhibitor of the ataxia-telangiectasia mutated kinase ATM. Cancer Res. 2004;64:9152–9. 10.1158/0008-5472.CAN-04-2727. 15604286

[18] Shu J, Wang X, Yang X et al. ATM inhibitor KU60019 synergistically sensitizes lung cancer cells to topoisomerase II poisons by multiple mechanisms. Sci Rep. 2023;13:882. 10.1038/s41598-023-28185-z. 36650267 PMC9845372

[19] Golding SE, Rosenberg E, Valerie N et al. Improved ATM kinase inhibitor KU-60019 radiosensitizes glioma cells, compromises insulin, AKT and ERK prosurvival signaling, and inhibits migration and invasion. Mol Cancer Ther. 2009;8:2894–902. 10.1158/1535-7163.MCT-09-0519. 19808981 PMC2761990

[20] Vecchio D, Daga A, Carra E et al. Predictability, efficacy and safety of radiosensitization of glioblastoma-initiating cells by the ATM inhibitor KU-60019. Int J Cancer. 2014;135:479–91. 10.1002/ijc.28680. 24443327

[21] Reinecke M, Ruprecht B, Poser S et al. Chemoproteomic selectivity profiling of PIKK and PI3K kinase inhibitors. ACS Chem Biol. 2019;14:655–64. 10.1021/acschembio.8b01020. 30901187

[22] Gill SJ, Wijnhoven PWG, Fok JHL et al. Radiopotentiation profiling of multiple inhibitors of the DNA damage response for early clinical development. Mol Cancer Ther. 2021;20:1614–26. 10.1158/1535-7163.MCT-20-0502. 34158341 PMC8650722

[23] Riches LC, Trinidad AG, Hughes G et al. Pharmacology of the ATM inhibitor AZD0156: potentiation of irradiation and olaparib responses preclinically. Mol Cancer Ther. 2020;19:13–25. 10.1158/1535-7163.MCT-18-1394. 31534013

[24] Davis SL, Hartman SJ, Bagby SM et al. ATM kinase inhibitor AZD0156 in combination with irinotecan and 5-fluorouracil in preclinical models of colorectal cancer. BMC Cancer. 2022;22:1107. 10.1186/s12885-022-10084-7. 36309653 PMC9617348

[25] Durant ST, Zheng L, Wang Y et al. The brain-penetrant clinical ATM inhibitor AZD1390 radiosensitizes and improves survival of preclinical brain tumor models. Sci Adv. 2018;4:eaat1719. 10.1126/sciadv.aat1719. 29938225 PMC6010333

[26] Jucaite A, Stenkrona P, Cselényi Z et al. Brain exposure of the ATM inhibitor AZD1390 in humans—a positron emission tomography study. Neuro Oncol. 2021;23:687–96. 10.1093/neuonc/noaa238. 33123736 PMC8041329

[27] Rainey MD, Charlton ME, Stanton RV et al. Transient inhibition of ATM kinase is sufficient to enhance cellular sensitivity to ionizing radiation. Cancer Res. 2008;68:7466–74. 10.1158/0008-5472.CAN-08-0763. 18794134 PMC2559948

[28] Tew BY, Kalfa AJ, Yang Z et al. ATM-inhibitor AZD1390 is a radiosensitizer for breast cancer CNS metastasis. Clin Cancer Res. 2023;29:4492–503. 10.1158/1078-0432.CCR-23-0290. 37585496 PMC10618650

[29] Wen P, Yang JT, Imber BS et al. Ctim-16. SAFETY and preliminary efficacy of Azd1390 + radiation therapy for glioblastoma. Neuro Oncol. 2024;26:viii88. 10.1093/neuonc/noae165.0349.

[30] Hu M, Zhou M, Bao X et al. ATM inhibition enhances cancer immunotherapy by promoting mtDNA leakage and cGAS/STING activation. J Clin Invest. 2021;131:e139333. 10.1172/JCI139333. 33290271 PMC7843232

[31] Grundy MK, Buckanovich RJ, Bernstein KA. Regulation and pharmacological targeting of RAD51 in cancer. NAR Cancer. 2020;2:zcaa024. 10.1093/narcan/zcaa024. 33015624 PMC7520849

[32] Klein HL . The consequences of Rad51 overexpression for normal and tumor cells. DNA Repair. 2008;7:686–93. 10.1016/j.dnarep.2007.12.008. 18243065 PMC2430071

[33] Bagnolini G, Milano D, Manerba M et al. Synthetic lethality in pancreatic cancer: discovery of a new RAD51–BRCA2 small molecule disruptor that inhibits homologous recombination and synergizes with Olaparib. J Med Chem. 2020;63:2588–619. 10.1021/acs.jmedchem.9b01526. 32037829 PMC7997579

[34] Nomme J, Renodon-Cornière A, Asanomi Y et al. Design of potent inhibitors of Human RAD51 recombinase based on BRC motifs of BRCA2 protein: modeling and experimental validation of a Chimera peptide. J Med Chem. 2010;53:5782–91. 10.1021/jm1002974. 20684611 PMC2917172

[35] Wang Z, Jia R, Wang L et al. The emerging roles of Rad51 in cancer and its potential as a therapeutic target. Front Oncol. 2022;12:935593. 35875146 10.3389/fonc.2022.935593 PMC9300834

[36] Huang F, Motlekar NA, Burgwin CM et al. Identification of specific inhibitors of human RAD51 recombinase using high-throughput screening. ACS Chem Biol. 2011;6:628–35. 10.1021/cb100428c. 21428443 PMC3117970

[37] Shkundina IS, Gall AA, Dick A et al. New RAD51 inhibitors to target homologous recombination in Human cells. Genes. 2021;12:920. 10.3390/genes12060920. 34208492 PMC8235719

[38] Gu P, Xue L, Zhao C et al. Targeting the homologous recombination pathway in cancer with a novel class of RAD51 inhibitors. Front Oncol. 2022;12:885186. 35646698 10.3389/fonc.2022.885186 PMC9136011

[39] Huang F, Mazin AV. A small molecule inhibitor of human RAD51 potentiates breast cancer cell killing by therapeutic agents in mouse xenografts. PLoS One. 2014;9:e100993. 10.1371/journal.pone.0100993. 24971740 PMC4074124

[40] Budke B, Logan HL, Kalin JH et al. RI-1: a chemical inhibitor of RAD51 that disrupts homologous recombination in human cells. Nucleic Acids Res. 2012;40:7347–57. 10.1093/nar/gks353. 22573178 PMC3424541

[41] Chen Q, Cai D, Li M et al. The homologous recombination protein RAD51 is a promising therapeutic target for cervical carcinoma. Oncol Rep. 2017;38:767–74. 10.3892/or.2017.5724. 28627709 PMC5561999

[42] King HO, Brend T, Payne HL et al. RAD51 Is a selective DNA repair target to radiosensitize glioma stem cells. Stem Cell Rep. 2017;8:125–39. 10.1016/j.stemcr.2016.12.005. PMC5233453 28076755

[43] Budke B, Kalin JH, Pawlowski M et al. An optimized RAD51 inhibitor that disrupts homologous recombination without requiring Michael acceptor reactivity. J Med Chem. 2013;56:254–63. 10.1021/jm301565b. 23231413 PMC3619390

[44] Fanning E, Klimovich V, Nager AR. A dynamic model for replication protein A (RPA) function in DNA processing pathways. Nucleic Acids Res. 2006;34:4126–37. 10.1093/nar/gkl550. 16935876 PMC1616954

[45] Zou Y, Liu Y, Wu X et al. Functions of human replication protein A (RPA): from DNA replication to DNA damage and stress responses. J Cell Physiol. 2006;208:267–73. 10.1002/jcp.20622. 16523492 PMC3107514

[46] Maréchal A, Zou L. RPA-coated single-stranded DNA as a platform for post-translational modifications in the DNA damage response. Cell Res. 2015;25:9–23. 10.1038/cr.2014.147. 25403473 PMC4650586

[47] Toledo LI, Altmeyer M, Rask M-B et al. ATR prohibits replication catastrophe by preventing global exhaustion of RPA. Cell. 2013;155:1088–103. 10.1016/j.cell.2013.10.043. 24267891

[48] Haring SJ, Mason AC, Binz SK et al. Cellular functions of human RPA1: multiple roles of domains in replication, repair, and checkpoints*. J Biol Chem. 2008;283:19095–111. 10.1074/jbc.M800881200. 18469000 PMC2441558

[49] VanderVere-Carozza PS, Gavande NS, Jalal SI et al. *In vivo* targeting replication protein A for cancer therapy. Front Oncol. 2022;12:826655 10.3389/fonc.2022.826655. 35251993 PMC8895377

[50] Jordan MR, Oakley GG, Mayo LD et al. The effect of replication protein A inhibition and post-translational modification on ATR kinase signaling. Sci Rep. 2024;14:19791. 10.1038/s41598-024-70589-y. 39187637 PMC11347632

[51] Glanzer JG, Liu S, Oakley GG. Small molecule inhibitor of the RPA70 N-terminal protein interaction domain discovered using *in silico* and *in vitro* methods. Bioorg Med Chem. 2011;19:2589–95. 10.1016/j.bmc.2011.03.012. 21459001 PMC3399738

[52] Glanzer JG, Carnes KA, Soto P et al. A small molecule directly inhibits the p53 transactivation domain from binding to replication protein A. Nucleic Acids Res. 2013;41:2047–59. 10.1093/nar/gks1291. 23267009 PMC3561959

[53] Glanzer JG, Liu S, Wang L et al. RPA inhibition increases replication stress and suppresses tumor growth. Cancer Res. 2014;74:5165–72. 10.1158/0008-5472.CAN-14-0306. 25070753 PMC4201622

[54] Dueva R, Krieger LM, Li F et al. Chemical inhibition of RPA by HAMNO alters cell cycle dynamics by impeding DNA replication and G2-to-M transition but has little effect on the radiation-induced DNA damage response. Int J Mol Sci. 2023;24:14941. 10.3390/ijms241914941. 37834389 PMC10573259

[55] Dueva R, Iliakis G. Replication protein A: a multifunctional protein with roles in DNA replication, repair and beyond. NAR Cancer. 2020;2:zcaa022. 10.1093/narcan/zcaa022. 34316690 PMC8210275

[56] Shuck SC, Turchi JJ. Targeted inhibition of replication protein A reveals cytotoxic activity, synergy with chemotherapeutic DNA-damaging agents, and insight into cellular function. Cancer Res. 2010;70:3189–98. 10.1158/0008-5472.CAN-09-3422. 20395205 PMC2882864

[57] Shuck S, Turchi J. Abstract #5545: the effect of a small molecule inhibitor of Replication Protein A (TDRL-505) on DNA binding, cellular function and platinum sensitivity. Cancer Res. 2009;69:5545.

[58] Mishra AK, Dormi SS, Turchi AM et al. Chemical inhibitor targeting the replication protein A–DNA interaction increases the efficacy of Pt-based chemotherapy in lung and ovarian cancer. Biochem Pharmacol. 2015;93:25–33. 10.1016/j.bcp.2014.10.013. 25449597 PMC4285624

[59] Hanada K, Budzowska M, Davies SL et al. The structure-specific endonuclease Mus81 contributes to replication restart by generating double-strand DNA breaks. Nat Struct Mol Biol. 2007;14:1096–104. 10.1038/nsmb1313. 17934473

[60] Minocherhomji S, Ying S, Bjerregaard VA et al. Replication stress activates DNA repair synthesis in mitosis. Nature. 2015;528:286–90. 10.1038/nature16139. 26633632

[61] Yin Y, Liu W, Shen Q et al. The DNA endonuclease Mus81 regulates ZEB1 expression and serves as a target of BET4 inhibitors in gastric cancer. Mol Cancer Ther. 2019;18:1439–50. 10.1158/1535-7163.MCT-18-0833. 31142662 PMC8345820

[62] Wang T, Zhang P, Li C et al. MUS81 inhibition enhances the anticancer efficacy of Talazoparib by impairing ATR/CHK1 signaling pathway in gastric cancer. Front Oncol. 2022;12:844135. 35480096 10.3389/fonc.2022.844135 PMC9035870

[63] Collie GW, Börjesson U, Chen Y et al. Fragment-based discovery of novel MUS81 inhibitors. ACS Med Chem Lett. 2024;15:1151–8. 10.1021/acsmedchemlett.3c00453. 39015284 PMC11247637

[64] Amarsanaa E, Wie M, Shin U et al. Synergistic enhancement of PARP inhibition via small molecule UNI66-mediated suppression of BRD4-dependent transcription of RAD51 and CtIP. NAR Cancer. 2025;7:zcaf013. 10.1093/narcan/zcaf013. 40308947 PMC12041917

[65] Hamilton EP, Wang JS, Oza AM et al. First-in-human study of AZD5153, A small-molecule inhibitor of bromodomain protein 4, in patients with relapsed/refractory malignant solid tumors and lymphoma. Mol Cancer Ther. 2023;22:1154–65. 10.1158/1535-7163.MCT-23-0065. 37486983 PMC10544002

[66] Lin C-H, Kuo JC-T, Li D et al. AZD5153, a bivalent BRD4 inhibitor, suppresses hepatocarcinogenesis by altering BRD4 chromosomal landscape and modulating the transcriptome of HCC cells. Front Cell Dev Biol. 2022;10:853652. 35399501 10.3389/fcell.2022.853652 PMC8987780

[67] Kang S, Yoo J, Myung K. PCNA cycling dynamics during DNA replication and repair in mammals. Trends Genet. 2024;40:526–39. 10.1016/j.tig.2024.02.006. 38485608

[68] Naryzhny SN, Lee H. Proliferating cell nuclear antigen in the cytoplasm interacts with components of glycolysis and cancer. FEBS Lett. 2010;584:4292–8. 10.1016/j.febslet.2010.09.021. 20849852

[69] Gu L, Lingeman R, Yakushijin F et al. The anticancer activity of a first-in-class small-molecule targeting PCNA. Clin Cancer Res. 2018;24:6053–65. 10.1158/1078-0432.CCR-18-0592. 29967249 PMC6279569

[70] Zhao H, Lo Y-H, Ma L et al. Targeting tyrosine phosphorylation of PCNA inhibits prostate cancer growth. Mol Cancer Ther. 2011;10:29–36. 10.1158/1535-7163.MCT-10-0778. 21220489 PMC3066081

[71] Gu L, Li M, Li CM et al. Small molecule targeting of transcription-replication conflict for selective chemotherapy. Cell Chem Biol. 2023;30:1235–47. 10.1016/j.chembiol.2023.07.001. 37531956 PMC10592352

[72] Bannoura SF, Khan HY, Uddin MH et al. Abstract 6876: a novel PCNA inhibitor AOH1996 demonstrates pre-clinical efficacy in pancreatic ductal adenocarcinoma models. Cancer Res. 2025;85:6876. 10.1158/1538-7445.AM2025-6876.

[73] Lingeman RG, Hickey R, Malkas L et al. Abstract 3864: AOH1996: a multi-faceted inhibitor of metastasis via PCNA and tumor microenvironment targeting. Cancer Res. 2025;85:3864. 10.1158/1538-7445.AM2025-3864.

[74] Lemech CR, Kichenadasse G, Marschner J-P et al. ATX-101, a cell-penetrating protein targeting PCNA, can be safely administered as intravenous infusion in patients and shows clinical activity in a phase 1 study. Oncogene. 2023;42:541–4. 10.1038/s41388-022-02582-6. 36564469 PMC9918429

[75] Sebesta M, Cooper CDO, Ariza A et al. Structural insights into the function of ZRANB3 in replication stress response. Nat Commun. 2017;8:15847. 10.1038/ncomms15847. 28621305 PMC5481773

[76] Gravina GL, Colapietro A, Mancini A et al. ATX-101, a peptide targeting PCNA, has antitumor efficacy alone or in combination with radiotherapy in murine models of human glioblastoma. Cancers. 2022;14:289. 10.3390/cancers14020289. 35053455 PMC8773508

[77] Krogh Søgaard C, Blindheim A, Røst LM et al. “Two hits - one stone”; increased efficacy of cisplatin-based therapies by targeting PCNA’s role in both DNA repair and cellular signaling. Oncotarget. 2018;9:32448–65. 30197755 10.18632/oncotarget.25963 PMC6126690

[78] García-Santisteban I, Peters GJ, Giovannetti E et al. USP1 deubiquitinase: cellular functions, regulatory mechanisms and emerging potential as target in cancer therapy. Mol Cancer. 2013;12:91. 10.1186/1476-4598-12-91. 23937906 PMC3750636

[79] Nijman SMB, Huang TT, Dirac AMG et al. The deubiquitinating enzyme USP1 regulates the fanconi anemia pathway. Mol Cell. 2005;17:331–9. 10.1016/j.molcel.2005.01.008. 15694335

[80] Liang F, Miller AS, Longerich S et al. DNA requirement in FANCD2 deubiquitination by USP1–UAF1–RAD51AP1 in the Fanconi anemia DNA damage response. Nat Commun. 2019;10:2849. 10.1038/s41467-019-10408-5. 31253762 PMC6599204

[81] Cadzow L, Brenneman J, Tobin E et al. The USP1 inhibitor KSQ-4279 overcomes PARP inhibitor resistance in homologous recombination–Deficient tumors. Cancer Res. 2024;84:3419–34. 10.1158/0008-5472.CAN-24-0293. 39402989 PMC11474170

[82] Liang Q, Dexheimer TS, Zhang P et al. A selective USP1–UAF1 inhibitor links deubiquitination to DNA damage responses. Nat Chem Biol. 2014;10:298–304. 10.1038/nchembio.1455. 24531842 PMC4144829

[83] Sun Y, Sha B, Huang W et al. ML323, a USP1 inhibitor triggers cell cycle arrest, apoptosis and autophagy in esophageal squamous cell carcinoma cells. Apoptosis. 2022;27:545–60. 10.1007/s10495-022-01736-x. 35654870

[84] Xu X, Mei X, Han K et al. The deubiquitinating enzyme USP1 is auto-ubiquitinated and destabilized by ML323 in colorectal cancer cells. EJMO. 2023;7:174–9.

[85] Rennie ML, Gundogdu M, Arkinson C et al. Structural and biochemical insights into the mechanism of action of the clinical USP1 inhibitor, KSQ-4279. J Med Chem. 2024;67:15557–68. 10.1021/acs.jmedchem.4c01184. 39190802 PMC11403619

[86] Yap TA, Lakhani NJ, Patnaik A et al. First-in-human phase I trial of the oral first-in-class ubiquitin specific peptidase 1 (USP1) inhibitor KSQ-4279 (KSQi), given as single agent (SA) and in combination with olaparib (OLA) or carboplatin (CARBO) in patients (pts) with advanced solid tumors, enriched for deleterious homologous recombination repair (HRR) mutations. J Clin Oncol. 2024;42:3005.

[87] Cadzow L, Tobin E, Sullivan P et al. Abstract ND01: KSQ-4279: A first-in-class USP1 inhibitor for the treatment of cancers with homologous recombination deficiencies. Cancer Res. 2022;82:ND01. 10.1158/1538-7445.AM2022-ND01.

[88] Wang J, Chen Y, Chen J et al. Abstract 670: preclinical candidate LAE120, a novel selective USP1 inhibitor shows effective anticancer and combination activity with PARP inhibitor. Cancer Res. 2024;84:670. 10.1158/1538-7445.AM2024-670.

[89] Li Y, Wu J, Liu J et al. Abstract 502: ISM3091, a novel selective USP1 inhibitor as a targeted anticancer therapy. Cancer Res. 2023;83:502. 10.1158/1538-7445.AM2023-502.

[90] Reaper PM, Griffiths MR, Long JM et al. Selective killing of ATM- or p53-deficient cancer cells through inhibition of ATR. Nat Chem Biol. 2011;7:428–30. 10.1038/nchembio.573. 21490603

[91] Sanjiv K, Hagenkort A, Calderón-Montaño JM et al. Cancer-specific synthetic lethality between ATR and CHK1 kinase activities. Cell Rep. 2016;17:3407–16. 10.1016/j.celrep.2016.12.031. PMC5638787 28009306

[92] Murga M, Campaner S, Lopez-Contreras AJ et al. Exploiting oncogene-induced replicative stress for the selective killing of Myc-driven tumors. Nat Struct Mol Biol. 2011;18:1331–5. 10.1038/nsmb.2189. 22120667 PMC4894468

[93] Yano K, Shiotani B. Emerging strategies for cancer therapy by ATR inhibitors. Cancer Sci. 2023;114:2709–21. 10.1111/cas.15845. 37189251 PMC10323102

[94] Williamson CT, Miller R, Pemberton HN et al. ATR inhibitors as a synthetic lethal therapy for tumours deficient in ARID1A. Nat Commun. 2016;7:13837. 10.1038/ncomms13837. 27958275 PMC5159945

[95] Shapiro GI, Wesolowski R, Devoe C et al. Phase 1 study of the ATR inhibitor berzosertib in combination with cisplatin in patients with advanced solid tumours. Br J Cancer. 2021;125:520–7. 10.1038/s41416-021-01406-w. 34040174 PMC8367944

[96] Thomas A, Redon CE, Sciuto L et al. Phase I study of ATR inhibitor M6620 in combination with Topotecan in patients with advanced solid tumors. JCO. 2018;36:1594–602. 10.1200/JCO.2017.76.6915. PMC5978471 29252124

[97] Jossé R, Martin SE, Guha R et al. ATR inhibitors VE-821 and VX-970 sensitize cancer cells to topoisomerase I inhibitors by disabling DNA replication initiation and fork elongation responses. Cancer Res. 2014;74:6968–79. 25269479 10.1158/0008-5472.CAN-13-3369 PMC4252598

[98] Middleton MR, Dean E, Evans TRJ et al. Phase 1 study of the ATR inhibitor berzosertib (formerly M6620, VX-970) combined with gemcitabine ± cisplatin in patients with advanced solid tumours. Br J Cancer. 2021;125:510–9. 10.1038/s41416-021-01405-x. 34040175 PMC8368196

[99] Takahashi N, Hao Z, Villaruz LC et al. Berzosertib plus Topotecan vs Topotecan alone in patients with relapsed small cell lung cancer: a randomized clinical trial. JAMA Oncol. 2023;9:1669–77. 10.1001/jamaoncol.2023.4025. 37824137 PMC10570917

[100] Gorecki L, Andrs M, Rezacova M et al. Discovery of ATR kinase inhibitor berzosertib (VX-970, M6620): clinical candidate for cancer therapy. Pharmacol Ther. 2020;210:107518. 10.1016/j.pharmthera.2020.107518. 32109490

[101] Foote KM, Nissink JWM, McGuire T et al. Discovery and characterization of AZD6738, a potent inhibitor of Ataxia telangiectasia mutated and Rad3 related (ATR) kinase with application as an anticancer agent. J. Med. Chem. 2018;61:9889–907. 10.1021/acs.jmedchem.8b01187. 30346772

[102] Kiesel BF, Guo J, Parise RA et al. Dose-dependent bioavailability and tissue distribution of the ATR inhibitor AZD6738 (ceralasertib) in mice. Cancer Chemother Pharmacol. 2022;89:231–42. 10.1007/s00280-021-04388-x. 35066692 PMC8829872

[103] Yap TA, Krebs MG, Postel-Vinay S et al. Ceralasertib (AZD6738), an oral ATR kinase inhibitor, in combination with carboplatin in patients with advanced solid tumors: a phase I study. Clin Cancer Res. 2021;27:5213–24. 10.1158/1078-0432.CCR-21-1032. 34301752 PMC9401487

[104] Kim ST, Smith SA, Mortimer P et al. Phase I study of Ceralasertib (AZD6738), a novel DNA damage repair agent, in combination with weekly Paclitaxel in refractory cancer. Clin Cancer Res. 2021;27:4700–9. 10.1158/1078-0432.CCR-21-0251. 33975862 PMC8974415

[105] Taniguchi H, Chakraborty S, Takahashi N et al. ATR inhibition activates cancer cell cGAS/STING-interferon signaling and promotes antitumor immunity in small-cell lung cancer. Sci Adv. 2024;10:eado4618. 10.1126/sciadv.ado4618. 39331709 PMC11430494

[106] Hailong S, Yan H, Yazhi X et al. ATR inhibitor AZD6738 enhances the antitumor activity of radiotherapy and immune checkpoint inhibitors by potentiating the tumor immune microenvironment in hepatocellular carcinoma. J Immunother Cancer. 2020;8:e000340. 32461345 10.1136/jitc-2019-000340 PMC7254123

[107] Kim R, Kwon M, An M et al. Phase II study of ceralasertib (AZD6738) in combination with durvalumab in patients with advanced/metastatic melanoma who have failed prior anti-PD-1 therapy. Ann Oncol. 2022;33:193–203. 10.1016/j.annonc.2021.10.009. 34710570

[108] Minsuk K, Gahyun K, Ryul K et al. Phase II study of ceralasertib (AZD6738) in combination with durvalumab in patients with advanced gastric cancer. J Immunother Cancer. 2022;10:e005041. 35790315 10.1136/jitc-2022-005041 PMC9258491

[109] Yap TA, Tan DSP, Terbuch A et al. First-in-Human trial of the oral Ataxia telangiectasia and RAD3-related (ATR) inhibitor BAY 1895344 in patients with advanced solid tumors. Cancer Discov. 2021;11:80–91. 10.1158/2159-8290.CD-20-0868. 32988960 PMC9554790

[110] Pusch FF, Dorado García H, Xu R et al. Elimusertib has antitumor activity in preclinical patient-derived pediatric solid tumor models. Mol Cancer Ther. 2024;23:507–19. 10.1158/1535-7163.MCT-23-0094. 38159110 PMC10985474

[111] Harold J, Bellone S, Manavella DD et al. Elimusertib (BAY1895344), a novel ATR inhibitor, demonstrates in vivo activity in ATRX mutated models of uterine leiomyosarcoma. Gynecol Oncol. 2023;168:157–65. 10.1016/j.ygyno.2022.11.014. 36442427 PMC9797429

[112] Stockton S, Shyr C, Cecchini M et al. A phase I study of ATR inhibitor BAY1895344 (elimusertib) plus topotecan (ETCTN 10402): results of dose escalation. JCO. 2024;42:3076. 10.1200/JCO.2024.42.16_suppl.3076.

[113] Yap TA, Tan DSP, Stathis A et al. Phase ib basket expansion trial and alternative-schedule dose-escalation study of ATR inhibitor elimusertib in advanced solid tumors with DNA damage response defects. Cancer Discov. 2025;15:2019–35. 10.1158/2159-8290.CD-24-1500. 40516108

[114] Jo U, Senatorov IS, Zimmermann A et al. Novel and highly potent ATR inhibitor M4344 kills cancer cells with replication stress, and enhances the chemotherapeutic activity of widely used DNA damaging agents. Mol Cancer Ther. 2021;20:1431–41. 10.1158/1535-7163.MCT-20-1026. 34045232 PMC9398135

[115] Yap TA, Fontana E, Lee EK et al. Camonsertib in DNA damage response-deficient advanced solid tumors: phase 1 trial results. Nat Med. 2023;29:1400–11. 10.1038/s41591-023-02399-0. 37277454 PMC10287555

[116] Black WC, Abdoli A, An X et al. Discovery of the potent and selective ATR inhibitor Camonsertib (RP-3500). J Med Chem. 2024;67:2349–68. 10.1021/acs.jmedchem.3c01917. 38299539

[117] Bakr A, Oing C, Köcher S et al. Involvement of ATM in homologous recombination after end resection and RAD51 nucleofilament formation. Nucleic Acids Res. 2015;43:3154–66. 10.1093/nar/gkv160. 25753674 PMC4381069

[118] Mann J, Niedermayer K, Krautstrunk J et al. Combined inhibition of RAD51 and CHK1 causes synergistic toxicity in cisplatin resistant cancer cells by triggering replication fork collapse. Int J Cancer. 2025;156:389–402. 10.1002/ijc.35164. 39239809 PMC11578078

[119] Lynch RC, Bendell JC, Advani RH et al. First-in-human phase I/II study of CYT-0851, a first-in-class inhibitor of RAD51-mediated homologous recombination in patients with advanced solid and hematologic cancers. JCO. 2021;39:3006. 10.1200/JCO.2021.39.15_suppl.3006.

[120] Guy JL, Maclay T, Day M et al. Abstract P2-05-05: RAD51 inhibition using CYT-0851, shows anti-cancer activity in cellular models of breast cancer and acts synergistically with PARP inhibitors. Cancer Res. 2020;80:P2–05-05. 10.1158/1538-7445.SABCS19-P2-05-05.

[121] Tsang ES, Munster PN. Targeting RAD51-mediated homologous recombination as a treatment for advanced solid and hematologic malignancies: opportunities and challenges ahead. OTT. 2022;15:1509–18. 10.2147/OTT.S322297. PMC9758980 36536949

[122] Castroviejo-Bermejo M, Cruz C, Llop-Guevara A et al. A RAD51 assay feasible in routine tumor samples calls PARP inhibitor response beyond BRCA mutation. EMBO Mol Med. 2018;10:e9172. 10.15252/emmm.201809172. 30377213 PMC6284440

[123] Feu S, Unzueta F, Ercilla A et al. RAD51 is a druggable target that sustains replication fork progression upon DNA replication stress. PLoS One. 2022;17:e0266645. 10.1371/journal.pone.0266645. 35969531 PMC9377619

[124] Bazan Russo TD, Mujacic C, Di Giovanni E et al. Polθ: emerging synthetic lethal partner in homologous recombination-deficient tumors. Cancer Gene Ther. 2024;31:1619–31. 10.1038/s41417-024-00815-2. 39122831 PMC11567890

[125] Wood RD, Doublié S. Genome protection by DNA polymerase θ. Annu Rev Genet. 2022;56:207–28. 10.1146/annurev-genet-072920-041046. 36028228 PMC10351424

[126] Mateos-Gomez PA, Kent T, Deng SK et al. The helicase domain of Polθ counteracts RPA to promote alt-NHEJ. Nat Struct Mol Biol. 2017;24:1116–23. 10.1038/nsmb.3494. 29058711 PMC6047744

[127] Zhou J, Gelot C, Pantelidou C et al. A first-in-class polymerase theta inhibitor selectively targets homologous-recombination-deficient tumors. Nat Cancer. 2021;2:598–610. 10.1038/s43018-021-00203-x. 34179826 PMC8224818

[128] Zatreanu D, Robinson HMR, Alkhatib O et al. Polθ inhibitors elicit BRCA-gene synthetic lethality and target PARP inhibitor resistance. Nat Commun. 2021;12:3636. 10.1038/s41467-021-23463-8. 34140467 PMC8211653

[129] Pismataro MC, Astolfi A, Barreca ML et al. Small molecules targeting DNA polymerase theta (POLθ) as promising synthetic lethal agents for precision cancer therapy. J Med Chem. 2023;66:6498–522. 10.1021/acs.jmedchem.2c02101. 37134182 PMC10226047

[130] Samnotra V, Moroz V, Shtessel L et al. Abstract CT169: first-in-human, phase 1/2 study of GSK4524101, an oral DNA polymerase theta inhibitor (POLQi), alone or combined with the poly(ADP-ribose) polymerase (PARP) inhibitor (PARPi) niraparib in adults with solid tumors. Cancer Res. 2024;84:CT169. 10.1158/1538-7445.AM2024-CT169.

[131] Meyer F, Becker S, Classen S et al. Prevention of DNA replication stress by CHK1 leads to chemoresistance despite a DNA repair defect in homologous recombination in breast cancer. Cells. 2020;9:238. 10.3390/cells9010238. 31963582 PMC7017274

[132] Yin Y, Lee WTC, Gupta D et al. A basal-level activity of ATR links replication fork surveillance and stress response. Mol Cell. 2021;81:4243–57. 10.1016/j.molcel.2021.08.009. 34473946 PMC8541912

[133] Knoblochova L, Duricek T, Vaskovicova M et al. CHK1-CDC25A-CDK1 regulate cell cycle progression and protect genome integrity in early mouse embryos. EMBO Rep. 2023;24:e56530. 10.15252/embr.202256530. 37694680 PMC10561370

[134] Hsu W-H, Zhao X, Zhu J et al. Checkpoint kinase 1 inhibition enhances cisplatin cytotoxicity and overcomes cisplatin resistance in SCLC by promoting mitotic cell death. J Thorac Oncol. 2019;14:1032–45. 10.1016/j.jtho.2019.01.028. 30771522 PMC6534433

[135] Ditano JP, Eastman A. Comparative activity and off-target effects in cells of the CHK1 inhibitors MK-8776, SRA737, and LY2606368. ACS Pharmacol Transl Sci. 2021;4:730–43. 10.1021/acsptsci.0c00201. 33860197 PMC8033610

[136] King C, Diaz HB, McNeely S et al. LY2606368 Causes replication catastrophe and antitumor effects through CHK1-dependent mechanisms. Mol Cancer Ther. 2015;14:2004–13. 10.1158/1535-7163.MCT-14-1037. 26141948

[137] Huang T-T, Brill E, Nair JR et al. Targeting the PI3K/mTOR pathway augments CHK1 inhibitor–induced replication stress and antitumor activity in high-grade serous ovarian cancer. Cancer Res. 2020;80:5380–92. 10.1158/0008-5472.CAN-20-1439. 32998994 PMC7718416

[138] Nair J, Huang T-T, Murai J et al. Resistance to the CHK1 inhibitor prexasertib involves functionally distinct CHK1 activities in BRCA wild-type ovarian cancer. Oncogene. 2020;39:5520–35. 10.1038/s41388-020-1383-4. 32647134 PMC7426265

[139] Brill E, Yokoyama T, Nair J et al. Prexasertib, a cell cycle checkpoint kinases 1 and 2 inhibitor, increases in vitro toxicity of PARP inhibition by preventing Rad51 foci formation in BRCA wild type high-grade serous ovarian cancer. Oncotarget. 2017;8:111026–40. 10.18632/oncotarget.22195. 29340034 PMC5762302

[140] Parmar K, Kochupurakkal BS, Lazaro J-B et al. The CHK1 inhibitor prexasertib exhibits monotherapy activity in high-grade serous ovarian cancer models and sensitizes to PARP inhibition. Clin Cancer Res. 2019;25:6127–40. 10.1158/1078-0432.CCR-19-0448. 31409614 PMC6801076

[141] Hong DS, Moore K, Patel M et al. Evaluation of Prexasertib, a checkpoint kinase 1 inhibitor, in a phase Ib study of patients with squamous cell carcinoma. Clin Cancer Res. 2018;24:3263–72. 10.1158/1078-0432.CCR-17-3347. 29643063 PMC6050086

[142] Lee J-M, Nair J, Zimmer A et al. Prexasertib, a cell cycle checkpoint kinase 1 and 2 inhibitor, in BRCA wild-type recurrent high-grade serous ovarian cancer: a first-in-class proof-of-concept phase 2 study. Lancet Oncol. 2018;19:207–15. 10.1016/S1470-2045(18)30009-3. 29361470 PMC7366122

[143] Zeng L, Nikolaev A, Xing C et al. CHK1/2 Inhibitor prexasertib suppresses NOTCH signaling and enhances cytotoxicity of cisplatin and radiation in head and neck squamous cell carcinoma. Mol Cancer Ther. 2020;19:1279–88. 10.1158/1535-7163.MCT-19-0946. 32371584

[144] Morimoto Y, Takada K, Takeuchi O et al. Prexasertib increases the sensitivity of pancreatic cancer cells to gemcitabine and S–1. Oncol Rep. 2020;43:689–99. 31789403 10.3892/or.2019.7421

[145] Giudice E, Huang T-T, Nair JR et al. The CHK1 inhibitor prexasertib in BRCA wild-type platinum-resistant recurrent high-grade serous ovarian carcinoma: a phase 2 trial. Nat Commun. 2024;15:2805. 10.1038/s41467-024-47215-6. 38555285 PMC10981752

[146] Hong D, Infante J, Janku F et al. Phase I study of LY2606368, a checkpoint kinase 1 inhibitor, in patients with advanced cancer. JCO. 2016;34:1764–71. 10.1200/JCO.2015.64.5788. PMC5321045 27044938

[147] Konstantinopoulos PA, Lee J-m, Gao B et al. A phase 2 study of prexasertib (LY2606368) in platinum resistant or refractory recurrent ovarian cancer. Gynecol Oncol. 2022;167:213–25. 10.1016/j.ygyno.2022.09.019. 36192237 PMC10673677

[148] Gatti-Mays ME, Karzai FH, Soltani SN et al. A phase II single arm pilot study of the CHK1 inhibitor prexasertib (LY2606368) in BRCA wild-type, advanced triple-negative breast cancer. Oncologist. 2020;25:1013–e1824. 10.1634/theoncologist.2020-0491. 32510664 PMC7938394

[149] Hong DS, Moore KN, Bendell JC et al. Preclinical evaluation and Phase Ib study of Prexasertib, a CHK1 inhibitor, and Samotolisib (LY3023414), a dual PI3K/mTOR inhibitor. Clin Cancer Res. 2021;27:1864–74. 10.1158/1078-0432.CCR-20-3242. 33495309

[150] Moore KN, Hong DS, Patel MR et al. A phase 1b trial of Prexasertib in combination with standard-of-care agents in advanced or metastatic cancer. Targ Oncol. 2021;16:569–89. 10.1007/s11523-021-00835-0. 34559360

[151] Lowery CD, Dowless M, Renschler M et al. Broad spectrum activity of the checkpoint kinase 1 inhibitor prexasertib as a single agent or chemopotentiator across a range of preclinical pediatric tumor models. Clin Cancer Res. 2019;25:2278–89. 10.1158/1078-0432.CCR-18-2728. 30563935 PMC6445779

[152] Li Q, Qian W, Zhang Y et al. A new wave of innovations within the DNA damage response. Sig Transduct Target Ther. 2023;8:338. 10.1038/s41392-023-01548-8. PMC10485079 37679326

[153] Chu QS, Jonker DJ, Provencher DM et al. A phase ib study of oral Chk1 inhibitor LY2880070 in combination with gemcitabine in patients with advanced or metastatic cancer. JCO. 2020;38:3581. 10.1200/JCO.2020.38.15_suppl.3581.

[154] Miller WH, Chu QS, Bouganim N et al. A phase ib study of oral Chk1 inhibitor LY2880070 as monotherapy in patients with advanced or metastatic cancer. JCO. 2020;38:3579. 10.1200/JCO.2020.38.15_suppl.3579.

[155] Huffman BM, Feng H, Parmar K et al. A phase I expansion cohort study evaluating the safety and efficacy of the CHK1 inhibitor LY2880070 with low-dose Gemcitabine in patients with metastatic pancreatic adenocarcinoma. Clin Cancer Res. 2023;29:5047–56. 10.1158/1078-0432.CCR-23-2005. 37819936 PMC10842136

[156] Chen X, Xu X, Chen Y et al. Structure of an activated DNA-PK and its implications for NHEJ. Mol Cell. 2021;81:801–10. 10.1016/j.molcel.2020.12.015. 33385326 PMC7897279

[157] Sibanda BL, Chirgadze DY, Ascher DB et al. DNA-PKcs structure suggests an allosteric mechanism modulating DNA double-strand break repair. Science. 2017;355:520–4. 10.1126/science.aak9654. 28154079

[158] Liu L, Chen X, Li J et al. Autophosphorylation transforms DNA-PK from protecting to processing DNA ends. Mol Cell. 2022;82:177–89. 10.1016/j.molcel.2021.11.025. 34936881 PMC8916119

[159] Fowler FC, Chen B-R, Zolnerowich N et al. DNA-PK promotes DNA end resection at DNA double strand breaks in G0 cells. eLife. 2022;11:e74700. 10.7554/eLife.74700. 35575473 PMC9122494

[160] Mladenov E, Fan X, Dueva R et al. Radiation-dose-dependent functional synergisms between ATM, ATR and DNA-PKcs in checkpoint control and resection in G2-phase. Sci Rep. 2019;9:8255. 10.1038/s41598-019-44771-6. 31164689 PMC6547644

[161] Gordhandas SB, Manning-Geist B, Henson C et al. Pre-clinical activity of the oral DNA-PK inhibitor, peposertib (M3814), combined with radiation in xenograft models of cervical cancer. Sci Rep. 2022;12:974. 10.1038/s41598-021-04618-5. 35046420 PMC8770623

[162] Kumar RJ, Chao HX, Simpson DA et al. Dual inhibition of DNA-PK and DNA polymerase theta overcomes radiation resistance induced by p53 deficiency. NAR Cancer. 2020;2:zcaa038. 10.1093/narcan/zcaa038. 33385162 PMC7751686

[163] Nakamura K, Karmokar A, Farrington PM et al. Inhibition of DNA-PK with AZD7648 sensitizes tumor cells to radiotherapy and induces type I IFN-dependent durable tumor control. Clin Cancer Res. 2021;27:4353–66. 10.1158/1078-0432.CCR-20-3701. 34011558 PMC9401489

[164] Carr MI, Chiu L-Y, Guo Y et al. DNA-PK inhibitor Peposertib amplifies radiation-induced inflammatory micronucleation and enhances tgfβ/PD-L1 targeted cancer immunotherapy. Mol Cancer Res. 2022;20:568–82. 10.1158/1541-7786.MCR-21-0612. 34980594 PMC9381110

[165] Dylgjeri E, Knudsen KE. DNA-PKcs: a targetable protumorigenic protein kinase. Cancer Res. 2022;82:523–33. 10.1158/0008-5472.CAN-21-1756. 34893509 PMC9306356

[166] Fok JHL, Ramos-Montoya A, Vazquez-Chantada M et al. AZD7648 is a potent and selective DNA-PK inhibitor that enhances radiation, chemotherapy and olaparib activity. Nat Commun. 2019;10:5065. 10.1038/s41467-019-12836-9. 31699977 PMC6838110

[167] Zhou Y, Börcsök J, Adib E et al. ATM deficiency confers specific therapeutic vulnerabilities in bladder cancer. Sci Adv. 2023;9:eadg2263. 10.1126/sciadv.adg2263. 37992168 PMC10664985

[168] Zenke FT, Zimmermann A, Sirrenberg C et al. Pharmacologic inhibitor of DNA-PK, M3814, potentiates radiotherapy and regresses Human tumors in mouse models. Mol Cancer Ther. 2020;19:1091–101. 10.1158/1535-7163.MCT-19-0734. 32220971

[169] Sun Q, Guo Y, Liu X et al. Therapeutic implications of p53 status on cancer cell fate following exposure to ionizing radiation and the DNA-PK inhibitor M3814. Mol Cancer Res. 2019;17:2457–68. 10.1158/1541-7786.MCR-19-0362. 31551253

[170] Samuels M, Falkenius J, Bar-Ad V et al. A phase 1 study of the DNA-PK inhibitor Peposertib in combination with radiation therapy with or without cisplatin in patients with advanced head and neck tumors. Int J Rad Oncol Biol Phys. 2024;118:743–56. 10.1016/j.ijrobp.2023.09.024. 37751793

[171] Romesser PB, Capdevila J, Garcia-Carbonero R et al. A Phase ib study of the DNA-PK inhibitor Peposertib combined with neoadjuvant chemoradiation in patients with locally advanced rectal cancer. Clin Cancer Res. 2024;30:695–702. 10.1158/1078-0432.CCR-23-1129. 38051750 PMC10870114

[172] Jo U, Arakawa Y, Zimmermann A et al. The novel ATR inhibitor Tuvusertib (M1774) induces replication protein overexpression and broad synergy with DNA-targeted anticancer drugs. Mol Cancer Ther. 2024;23:911–23. 10.1158/1535-7163.MCT-23-0402. 38466804 PMC11555614

[173] Perez B, Aljumaily R, Marron TU et al. Phase I study of peposertib and avelumab with or without palliative radiotherapy in patients with advanced solid tumors. ESMO Open. 2024;9:102217. 10.1016/j.esmoop.2023.102217. 38320431 PMC10937199

[174] O’Sullivan J, Tedim Ferreira M, Gagné J-P et al. Emerging roles of eraser enzymes in the dynamic control of protein ADP-ribosylation. Nat Commun. 2019;10:1182. 30862789 10.1038/s41467-019-08859-x PMC6414514

[175] Pascal JM, Ellenberger T. The rise and fall of poly(ADP-ribose): an enzymatic perspective. DNA Repair. 2015;32:10–6. 10.1016/j.dnarep.2015.04.008. 25963443 PMC4522361

[176] Paradkar S, Purcell J, Cui A et al. PARG inhibition induces nuclear aggregation of PARylated PARP1. Structure. 2024;32:2083–93. 10.1016/j.str.2024.09.006. 39406247

[177] Slade D . PARP and PARG inhibitors in cancer treatment. Genes Dev. 2020;34:360–94. 10.1101/gad.334516.119. 32029455 PMC7050487

[178] Ravindranathan R, Somuncu O, da Costa AABA et al. PARG inhibitor sensitivity correlates with accumulation of single-stranded DNA gaps in preclinical models of ovarian cancer. Proc Natl Acad Sci USA. 2024;121:e2413954121. 10.1073/pnas.2413954121. 39546575 PMC11588084

[179] Pillay N, Tighe A, Nelson L et al. DNA replication vulnerabilities render ovarian cancer cells sensitive to poly(ADP-Ribose) glycohydrolase inhibitors. Cancer Cell. 2019;35:519–33. 10.1016/j.ccell.2019.02.004. 30889383 PMC6428690

[180] Coulson-Gilmer C, Morgan RD, Nelson L et al. Replication catastrophe is responsible for intrinsic PAR glycohydrolase inhibitor-sensitivity in patient-derived ovarian cancer models. J Exp Clin Cancer Res. 2021;40:323. 10.1186/s13046-021-02124-0. 34656146 PMC8520217

[181] Abed M, Muñoz D, Seshadri V et al. Abstract 6093: IDE161, a potential first-in-class clinical candidate PARG inhibitor, selectively targets homologous-recombination-deficient and PARP inhibitor resistant breast and ovarian tumors. Cancer Res. 2023;83:6093. 10.1158/1538-7445.AM2023-6093.

[182] Holleran JP, Rodems TS, Sharma S et al. Abstract 2083: discovery of ETX-19477, a novel and selective PARG inhibitor with high potency against tumors with underlying replication stress. Cancer Res. 2024;84:2083. 10.1158/1538-7445.AM2024-2083.

[183] Pommier Y, Leo E, Zhang H et al. DNA topoisomerases and their poisoning by anticancer and antibacterial drugs. Chem Biol. 2010;17:421–33. 10.1016/j.chembiol.2010.04.012. 20534341 PMC7316379

[184] Gilbert DC, Chalmers AJ, El-Khamisy SF. Topoisomerase I inhibition in colorectal cancer: biomarkers and therapeutic targets. Br J Cancer. 2012;106:18–24. 10.1038/bjc.2011.498. 22108516 PMC3251848

[185] Zhao H, Rybak P, Dobrucki J et al. Relationship of DNA damage signaling to DNA replication following treatment with DNA topoisomerase inhibitors camptothecin/topotecan, mitoxantrone, or etoposide. Cytometry Pt A. 2012;81A:45–51. 10.1002/cyto.a.21172. PMC3242513 22140093

[186] Pommier Y . Topoisomerase I inhibitors: camptothecins and beyond. Nat Rev Cancer. 2006;6:789–802. 10.1038/nrc1977. 16990856

[187] Staker BL, Feese MD, Cushman M et al. Structures of three classes of anticancer agents bound to the Human topoisomerase I−DNA covalent complex. J Med Chem. 2005;48:2336–45. 10.1021/jm049146p. 15801827

[188] Tkaczuk KH, Zamboni WC, Tait NS et al. Phase I study of docetaxel and topotecan in patients with solid tumors. Cancer Chemother Pharmacol. 2000;46:442–8. 10.1007/s002800000180. 11138457

[189] Jones S, Thompson D, Barton J et al. A randomized phase II trial of oral Topotecan versus Docetaxel in the second-line treatment of non–small-cell lung cancer. Clin Lung Cancer. 2008;9:154–9. 10.3816/CLC.2008.n.023. 18621625

[190] Spigel DR, Hainsworth JD, Gandhi JG et al. A Phase II trial of carboplatin and weekly topotecan in the first-line treatment of patients with extensive stage small cell lung cancer. J Thorac Oncol. 2010;5:862–6. 10.1097/JTO.0b013e3181d86a4f. 20521352

[191] Tewari KS, Sill MW, Birrer MJ et al. Final survival analysis of topotecan and paclitaxel for first-line treatment of advanced cervical cancer: an NRG oncology randomized study. Gynecol Oncol. 2023;171:141–50. 10.1016/j.ygyno.2023.01.010. 36898292 PMC10286827

[192] Ocean AJ, Niedzwiecki D, Atkins JN et al. LE-SN38 for metastatic colorectal cancer after progression on oxaliplatin: results of CALGB 80402. JCO. 2008;26:4109. 10.1200/jco.2008.26.15_suppl.4109.

[193] Lee Y-M, Chen Y-H, Ou D-L et al. SN-38, an active metabolite of irinotecan, enhances anti-PD-1 treatment efficacy in head and neck squamous cell carcinoma. J Pathol. 2023;259:428–40. 10.1002/path.6055. 36641765

[194] Lipsyc-Sharf M, Ou F-S, Yurgelun MB et al. Cetuximab and Irinotecan with or without Bevacizumab in refractory metastatic colorectal cancer: BOND-3, an ACCRU network randomized clinical trial. Oncologist. 2022;27:292–8. 10.1093/oncolo/oyab025. 35380713 PMC8982431

[195] Middleton G, Brown S, Lowe C et al. A randomised phase III trial of the pharmacokinetic biomodulation of irinotecan using oral ciclosporin in advanced colorectal cancer: results of the Panitumumab, Irinotecan & Ciclosporin in COLOrectal cancer therapy trial (PICCOLO). Eur J Cancer. 2013;49:3507–16. 10.1016/j.ejca.2013.06.017. 23953030

[196] Wang Y-T, Ji W-D, Jiao H-M et al. Targeting 4-1BB for tumor immunotherapy from bench to bedside. Front Immunol. 2022;13:975926. 36189243 10.3389/fimmu.2022.975926 PMC9523430

[197] Pratz KW, Rudek MA, Gojo I et al. A phase I study of topotecan, carboplatin and the PARP inhibitor veliparib in acute leukemias, aggressive myeloproliferative neoplasms, and chronic myelomonocytic leukemia. Clin Cancer Res. 2017;23:899–907. 10.1158/1078-0432.CCR-16-1274. 27551000 PMC5315611

[198] Thomas A, Fontaine SD, Diolaiti ME et al. PLX038: a long-acting topoisomerase I inhibitor with robust antitumor activity in ATM-deficient tumors and potent synergy with PARP inhibitors. Mol Cancer Ther. 2022;21:1722–8. 10.1158/1535-7163.MCT-22-0217. 35999657 PMC10673686

[199] Hendrickson AEW, Foster NR, Clasemann T et al. Phase II clinical trial of PLX038 in patients with platinum resistant ovarian, primary peritoneal and fallopian tube cancer. JCO. 2024;42:TPS5630. 10.1200/JCO.2024.42.16_suppl.TPS5630.

[200] Pacenta HL, Allen-Rhoades W, Langenau D et al. Prioritization of novel agents for patients with rhabdomyosarcoma: a report from the children’s oncology group (COG) New Agents for rhabdomyosarcoma Task Force. JCM. 2021;10:1416. 10.3390/jcm10071416. 33915882 PMC8037615

[201] Chan EM, Shibue T, McFarland JM et al. WRN helicase is a synthetic lethal target in microsatellite unstable cancers. Nature. 2019;568:551–6. 10.1038/s41586-019-1102-x. 30971823 PMC6580861

[202] Wang M, Ran X, Leung W et al. ATR inhibition induces synthetic lethality in mismatch repair-deficient cells and augments immunotherapy. Genes Dev. 2023;37:929–43. 10.1101/gad.351084.123. 37932012 PMC10691477

[203] Hao S, Tong J, Jha A et al. Synthetical lethality of Werner helicase and mismatch repair deficiency is mediated by p53 and PUMA in colon cancer. Proc Natl Acad Sci USA. 2022;119:e2211775119. 10.1073/pnas.2211775119. 36508676 PMC9907101

[204] Zong D, Koussa NC, Cornwell JA et al. Comprehensive mapping of cell fates in microsatellite unstable cancer cells supports dual targeting of WRN and ATR. Genes Dev. 2023;37:913–28. 10.1101/gad.351085.123. 37932011 PMC10691471

[205] Ferretti S, Hamon J, de Kanter R et al. Discovery of WRN inhibitor HRO761 with synthetic lethality in MSI cancers. Nature. 2024;629:443–9. 10.1038/s41586-024-07350-y. 38658754 PMC11078746

[206] Baltgalvis KA, Lamb KN, Symons KT et al. Chemoproteomic discovery of a covalent allosteric inhibitor of WRN helicase. Nature. 2024;629:435–42. 10.1038/s41586-024-07318-y. 38658751

[207] Sui Q, Zhou Y, Li M et al. Design, synthesis, and structure–activity relationship studies of triazolo-pyrimidine derivatives as WRN inhibitors for the treatment of MSI tumors. Eur J Med Chem. 2025;282:117039. 10.1016/j.ejmech.2024.117039. 39561494

[208] Chen S, Wang Z, Cao Z et al. Targeting the Werner syndrome protein in microsatellite instability cancers: mechanisms and therapeutic potential. Clin Exp Med. 2025;25:278. 10.1007/s10238-025-01781-1. 40767886 PMC12328481

[209] Wie M, Khim KW, Groehler IV et al. Alkylation of nucleobases by 2-chloro-*N,N*-diethylethanamine hydrochloride (CDEAH) sensitizes PARP1-deficient tumors. NAR Cancer. 2023;5:zcad042. 10.1093/narcan/zcad042. 37554969 PMC10405566

[210] Kwon T, Ra JS, Lee S et al. Precision targeting tumor cells using cancer-specific InDel mutations with CRISPR–Cas9. Proc Natl Acad Sci USA. 2022;119:e2103532119. 10.1073/pnas.2103532119. 35217600 PMC8892319

